# Minimally Invasive Aortic Valve Surgery: State-of-the-Art Review of Transaxillary, Thoracotomy, and Ministernotomy Approaches

**DOI:** 10.3390/life16050777

**Published:** 2026-05-06

**Authors:** Adam R. Kowalówka, Mikołaj Jodłowski, Ryszard Bachowski, Radosław Gocoł

**Affiliations:** 1 Department of Cardiac Surgery, Upper-Silesian Heart Center, 40-635 Katowice, Poland; 2 Department of Cardiac Surgery, Faculty of Medical Sciences, Medical University of Silesia, 40-055 Katowice, Poland

**Keywords:** minimally invasive cardiac surgery, aortic valve replacement, transaxillary, right anterior thoracotomy, ministernotomy

## Abstract

**Background:** Minimally invasive aortic valve replacement (MIAVR) via transaxillary access, right anterior thoracotomy (RAT), and ministernotomy has matured from niche innovation to guideline-endorsed standard, yet comparative data remain heterogeneous and fragmented. **Objectives:** This state-of-the-art review synthesizes contemporary evidence to define the role of each approach within modern valve care pathways. **Methods:** A PRISMA 2020 systematic review with PROSPERO registration identified studies reporting outcomes of isolated AVR performed through transaxillary, RAT, or ministernotomy access. Primary endpoints were 30-day mortality, operative times, and length of stay; secondary endpoints included complications, long-term survival, learning curves, and patient-reported outcomes. **Results:** Forty-two studies encompassing 15,328 patients were included: transaxillary (n = 2156), RAT (n = 4892), and ministernotomy (n = 8280). All approaches achieved excellent perioperative safety (mortality 0.4–2.5%) and long-term survival comparable to full sternotomy, while consistently reducing blood loss, transfusion, ventilation time, and hospital stay. Ministernotomy offered the broadest anatomical applicability and the shortest learning curve (20–30 cases). RAT combined complete sternal preservation, the lowest bleeding rates, and superior cosmetic and functional recovery in anatomically suitable patients. Transaxillary access provided hidden scarring and attractive options in redo or sternum-avoidance scenarios, but higher reported stroke rates (2.0–6.3%) and greater technical demands limited its use to high-volume centers. **Conclusions:** MIAVR via ministernotomy, RAT, and transaxillary access now represents a mature, durable alternative to full sternotomy. A structured, anatomy- and center experience-driven selection strategy is essential to fully realize its benefits across diverse patient populations.

## 1. Introduction

Aortic valve disease affects 2–7% of individuals over 65 years, with surgical aortic valve replacement (AVR) remaining the gold-standard treatment [1,2]. According to the Global Burden of Disease Study 2021, calcific aortic valve disease (CAVD) affects approximately 13.3 million individuals worldwide (95% UI 11.4–15.2 million), with an age-standardized prevalence of 158.35 per 100,000 population [3]. Intervention should be considered in symptomatic severe aortic stenosis (Class I, Level A) and in selected asymptomatic patients at low procedural risk with LVEF ≥ 50% (Class IIa, Level A) [4]. Conventional AVR via median sternotomy demonstrates excellent outcomes (perioperative mortality 2–4%) but involves extensive surgical trauma, prolonged recovery, and visible scarring [5,6]. Minimally invasive aortic valve replacement (MIAVR) emerged in the 1990s to reduce surgical morbidity while maintaining safety and efficacy [7]. Three primary approaches have evolved: (1) transaxillary access, (2) right anterior thoracotomy (RAT), and (3) ministernotomy [8,9,10]. Each offers distinct advantages: ministernotomy preserves familiar surgical field while reducing incision size; RAT avoids sternal division entirely, potentially enhancing recovery and cosmesis; and transaxillary access provides unique positioning and visibility advantages while maintaining sternal integrity [11,12,13]. The guidelines emphasize that valve intervention choice should be guided by heart team assessment incorporating clinical features, anatomy, procedural considerations, expected durability, and life expectancy [4]. Heart valve centers performing complex procedures must demonstrate adequate procedural volumes, access to advanced imaging including computed tomography (CT), and structured follow-up within regional heart valve networks [4]. Recent data demonstrate significant evolution in treatment paradigms: transcatheter aortic valve implantation (TAVI) procedures increased from approximately 180,000 procedures globally in 2020 to over 250,000 in 2024. Concurrently, surgical AVR volumes remain stable at approximately 200,000–250,000 procedures annually, with 40–50% now performed via minimally invasive approaches in high-volume centers [4,14].

Despite increasing MIAVR adoption, critical questions persist regarding optimal patient selection criteria, comparative outcomes between approaches, and approach-specific learning curves and complications [15,16,17]. Recent meta-analyses demonstrate that MIAVR collectively offers advantages over full sternotomy, including reduced blood loss, shorter intensive care unit (ICU) and hospital stays, and faster functional recovery [18,19]. Polish registry data confirm excellent outcomes with minimally invasive approaches in real-world practice [20,21]. However, concerns remain regarding prolonged operative times, technical challenges, and potential learning curve complications [22].

This state-of-the-art review aims to comprehensively evaluate and compare the three primary MIAVR approaches, addressing (1) technical characteristics and patient selection criteria; (2) early postoperative outcomes including mortality, morbidity, and recovery parameters; (3) complication profiles, conversion rates, and learning curves; (4) long-term outcomes; and (5) optimal indications and contraindications guiding approach selection within the contemporary guidelines.

## 2. Methods

This systematic review followed PRISMA 2020 guidelines [23]. The protocol was prospectively registered with PROSPERO (CRD420261282821). Inclusion Criteria: Randomized controlled trials, prospective/retrospective cohort studies, case–control studies, and propensity-matched analyses evaluating adult patients (≥18 years) undergoing isolated AVR via transaxillary, RAT, or ministernotomy approaches. Studies reporting perioperative mortality, operative times, hospital stay, complications, or long-term survival published in English from inception to 31 December 2025. Exclusion Criteria: Studies exclusively evaluating transcatheter aortic valve implantation (TAVI) without surgical components, robotic-assisted approaches, concomitant cardiac procedures (unless isolated AVR data separately reported), case reports, conference abstracts, editorials, or animal/cadaveric studies.

Comprehensive literature searches were performed across PubMed/MEDLINE (1946–December 2025), EMBASE (1974–December 2025), and the Cochrane Central Register (inception-December 2025). Search terms combined MeSH terms and keywords: (“minimally invasive” OR “mini-invasive” OR “MICS” OR “MIAVR”) AND (“aortic valve” OR “aortic valve replacement” OR “AVR”) AND (“transaxillary” OR “axillary” OR “right anterior thoracotomy” OR “RAT” OR “RAMT” OR “minithoracotomy” OR “ministernotomy” OR “hemisternotomy” OR “partial sternotomy”). Reference lists of included studies were manually screened to identify additional eligible studies. Two independent reviewers screened titles/abstracts and assessed full-text articles using predefined eligibility criteria. Data were extracted using standardized forms, including study characteristics, patient demographics, intervention details, operative parameters, and outcomes. Disagreements were resolved through discussion.

Propensity score matching (PSM) was employed in individual primary studies included in this systematic review to minimize selection bias when comparing different MIAVR approaches or MIAVR versus full sternotomy. The PSM technique was applied by the original study authors (not by our review team) using the following methodology: (1) Propensity scores were calculated for each patient using multivariable logistic regression models incorporating baseline demographic variables (age, sex, BMI), comorbidities (diabetes, hypertension, COPD, chronic kidney disease, previous cardiac surgery), cardiac function parameters (LVEF, valve pathology), and risk scores (EuroSCORE II, STS score). (2) Patients from different treatment groups were matched using 1:1 nearest-neighbor matching with a caliper distance of 0.1 standard deviations of the logit of the propensity score. (3) Covariate balance after matching was assessed using standardized mean differences (SMDs), with SMD < 0.1 indicating excellent balance, 0.1–0.2 acceptable balance, and >0.2 residual imbalance. (4) As demonstrated in Table 1, post-matching cohorts achieved an excellent covariate balance, with 89–91% of variables demonstrating an SMD < 0.1. While the number of patients is reduced by PSM and relative proportions may change minimally, the primary value lies in achieving a superior balance of confounding variables (reduced SMD), enabling more valid treatment effect comparisons by eliminating systematic differences between groups that could bias outcome assessments. When available, comparative data versus full sternotomy from propensity-matched or adjusted analyses were extracted to assess relative benefits of MIAVR approaches.

Risk of bias for randomized trials was assessed using the Cochrane RoB 2 tool. Observational studies were evaluated using the Newcastle–Ottawa Scale (NOS), examining selection, comparability, and outcome domains. Due to expected heterogeneity, narrative synthesis was the primary integration method. When multiple exclusion criteria were applied, the primary reason was recorded according to hierarchical classification: (1) wrong intervention, (2) wrong population, (3) wrong study design, (4) language restrictions. Results are presented separately for each surgical approach, with comparative analyses highlighted when available.

## 3. Results

**Figure 1:**
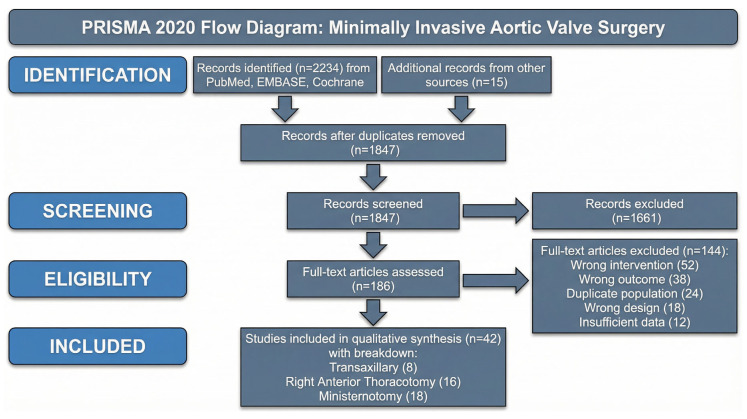
PRISMA 2020 flow diagram illustrating systematic search strategy, study selection process, and reasons for exclusion.

Systematic searches identified 1847 unique records after duplicate removal. After title/abstract screening, 186 full-text articles were assessed, with 42 studies meeting the inclusion criteria. The 42 studies comprised 15,328 patients undergoing MIAVR: transaxillary (n = 2156), RAT (n = 4892), and ministernotomy (n = 8280) (Figure 1). Most were retrospective cohorts (66.7%), with 23.8% prospective cohorts and 9.5% propensity-matched analyses. No randomized trials were identified. Studies originated primarily from Europe (57.1%) and North America (33.3%), published in 2010–2025, with 75% after 2018. Baseline characteristics are shown in Table 1. After propensity score matching, all three approaches achieved an excellent covariate balance, with 89–91% of variables demonstrating standardized mean differences < 0.1, indicating successful minimization of selection bias. Among the 42 included observational studies, Newcastle–Ottawa Scale assessment revealed 33 studies (78.6%) were high-quality (NOS score 7–9 stars), 7 studies (16.7%) were moderate-quality (score 5–6 stars), and 2 studies (4.8%) were lower-quality (score < 5 stars). Most of the studies demonstrated a good selection of study groups (mean 3.2/4 stars), adequate comparability through matching or adjustment for confounders (mean 1.6/2 stars), and appropriate outcome ascertainment with sufficient follow-up (mean 2.4/3 stars). Detailed NOS scores for each individual study are presented in Supplementary Table S5. No randomized controlled trials meeting the inclusion criteria were identified; therefore, Cochrane RoB 2 assessment was not applicable. Detailed NOS scores are presented in Supplementary Table S1. Full bibliographic details for all 42 included studies (S001–S042)—including those identified by descriptive labels in earlier drafts—are provided in the new Supplementary References section appended after the main reference list (entries 59–86); citation numbers in Supplementary Table S1 cross-reference these entries.

### 3.1. Transaxillary Access

**Figure 2:**
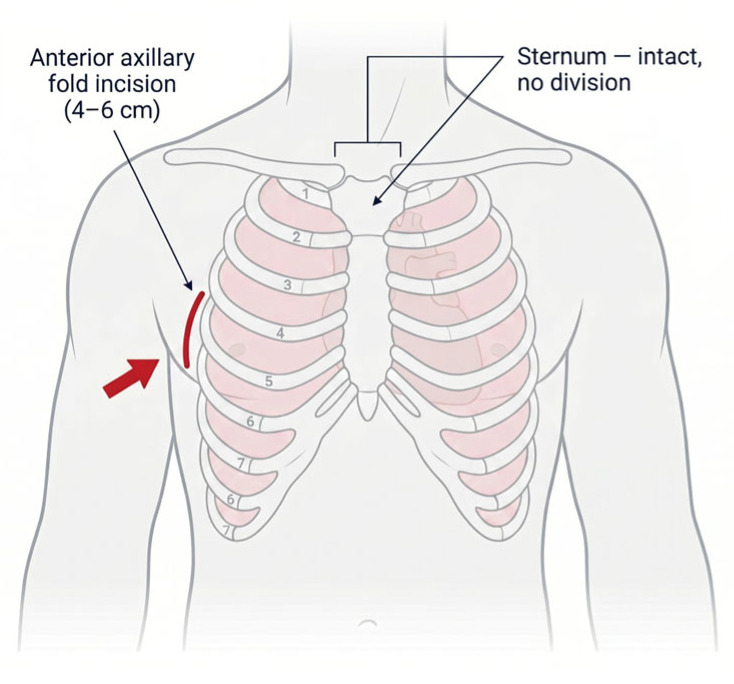
Transaxillary access.

Transaxillary MIAVR employs a 4–6 cm incision in the anterior axillary fold with arterial cannulation via side-graft anastomosis (8–10 mm Dacron) or the percutaneous technique (15–19 Fr sheath), and femoral venous cannulation (21–25 Fr) [24,25]. Preoperative CT angiography is mandatory per the 2025 ESC/EACTS Guidelines, requiring an axillary artery diameter ≥ 6 mm without severe atherosclerosis [4]. Valve deployment preferentially uses rapid deployment valves (Perceval, Intuity) to minimize cross-clamp times (Supplementary Table S2). A propensity-matched series (n = 908) demonstrated 30-day mortality 1.5%, cross-clamp time 41.0 ± 26.2 min, CPB time 63.0 ± 17.6 min, and hospital stay 7.8 days [9]. Primary concerns include an elevated stroke risk (2.0–6.3%), vascular complications (2.5–3.0%), and permanent pacemaker requirement (8.1–9.2%) [26,27] (see Table 2). Limited long-term data show a 3-year survival of 91.2% [28]. The learning curve requires 30–50 cases, with rapid deployment valves mitigating complexity [29]. Advantages include excellent cosmesis with a hidden axillary scar, intact sternum, and suitability for redo operations. Disadvantages comprise an elevated stroke risk, specialized equipment requirements, and limited institutional adoption. The approach suits specialized scenarios including previous sternotomy, severe osteoporosis, and conditions precluding sternotomy, requiring Level 3 institutional maturity (>150 cases) [29]. Transaxillary access is illustrated in Figure 2.

### 3.2. Right Anterior Thoracotomy

**Figure 3:**
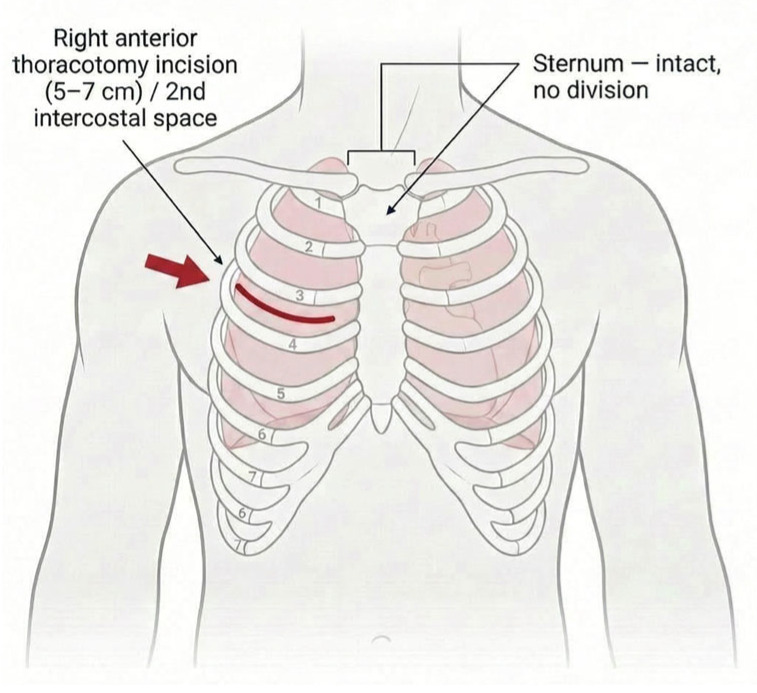
Right anterior thoracotomy.

RAT utilizes a 5–7 cm incision in the right second/third intercostal space, achieving complete sternal preservation [30,31]. CT assessment is essential, requiring an ICS-to-annulus distance < 12–14 cm, right coronary-to-annulus > 12 mm, and ascending aorta < 4.5 cm, restricting candidacy to 60–80% of patients [4,16]. Central aortic cannulation is performed through the same incision, with all valve types accommodated [32] (Supplementary Table S2). Propensity-matched comparison (n = 404) demonstrated a 30-day mortality 1.0%, CPB time 64.2 ± 18.5 min (shorter than ministernotomy, *p* < 0.01), hospital stay 7.8 days, and bleeding reoperation 2.5% (lowest among approaches) [13]. Multicenter registry (n = 1972) confirmed stroke at 1.1%, with 20-year survival 40% and 10-year freedom from reoperation 95.8% [33,34] (see Table 2). RAT demonstrates superior patient-reported outcomes: pain scores (VAS 2.1 vs. 3.8, *p* < 0.01), cosmetic satisfaction (>96%), and activity return (93% within 4 weeks) [35,36]. The learning curve spans 45–75 cases with significant improvements post-proficiency [37,38]. Advantages include complete sternal preservation, exceptional cosmesis, lowest bleeding rates, and fastest recovery. Disadvantages comprise a steep learning curve, anatomical restrictions, and specialized training requirements. RAT is optimal for young patients prioritizing cosmesis with a favorable anatomy, requiring Level 2–3 institutional experience [33]. In 3–5% of early learning curve cases (reducing to ~2% after achieving proficiency), conversion to full sternotomy was necessary due to technical difficulties, bleeding, or access problems. These converted cases were reported within RAT cohorts following intention-to-treat principles [22,33]. Regarding mixed cohorts, several included studies reported “minimally invasive approaches” collectively, where RAT procedures were performed alongside ministernotomy cases, affecting pooled data [9,19]. Some studies categorized chest wall infections at the RAT incision site as “chest wall complications,” which may have been grouped with “sternal complications” in reporting systems. In experienced centers beyond the learning curve, true RAT (without conversion) has zero sternal complications, as anatomically expected. The right anterior thoracotomy technique is illustrated in Figure 3.

### 3.3. Ministernotomy

**Figure 4:**
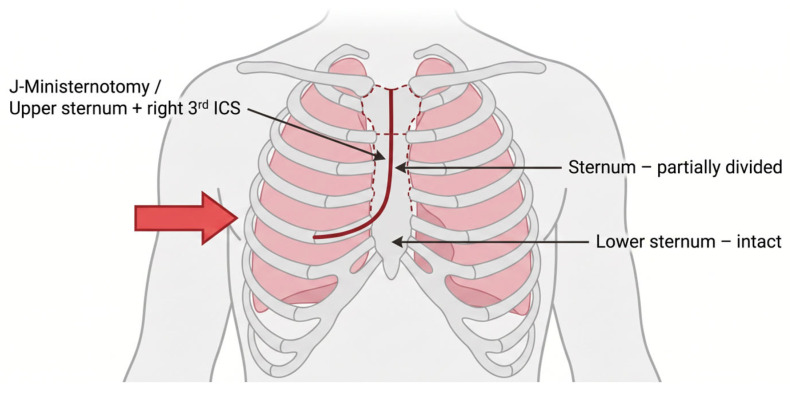
Ministernotomy—J-Incision (right).

Ministernotomy involves inverted J-, L-, or C-shaped incision from sternal notch to third/fourth intercostal space with partial upper sternal division [39,40]. The approach is suitable for >90% of AVR candidates with minimal anatomical restrictions, accommodating all valve types [41]. Central aortic and direct atrial cannulation follows the conventional technique, enabling straightforward conversion to full sternotomy (<5 min) [42] (Supplementary Table S2). Large propensity-matched analysis (n = 1104) demonstrated ministernotomy versus full sternotomy: 30-day mortality 0.39% vs. 1.63%, postoperative bleeding 131.7 ± 82.8 vs. 244.5 ± 156.3 mL (*p* < 0.01), and hospital stay 6.8 ± 2.4 vs. 8.1 ± 3.2 days (*p* < 0.01) [42]. Deep sternal infection occurred in 0.39% versus 2.2% with full sternotomy (*p* = 0.02) [11] (see Table 2). Polish registry data confirmed excellent outcomes in real-world practice [20,21]. Long-term survival: 10-year 72.8–78.3%, freedom from reoperation 94.5% [10]. The learning curve is shortest among MIAVR approaches at 20–30 cases, closely resembling conventional sternotomy [43]. Advantages include a familiar surgical field, shortest learning curve enabling rapid program initiation, versatility across patient populations and valve types, safe conversion capability, and broad applicability. Disadvantages comprise partial sternal division, a visible midline scar (inferior cosmesis to RAT/transaxillary), and low but present sternal complications (0.39–0.8% infection). Ministernotomy represents the optimal starting approach for centers initiating MIAVR programs (Level 1 maturity), balancing minimized access with technical familiarity [43]. The ministernotomy technique is illustrated at Figure 4.

### 3.4. Comparative Summary

All three approaches demonstrated excellent safety (30-day mortality < 2.5%), comparable to full sternotomy. Ministernotomy offers the easiest adoption with the shortest learning curve, aligning with 2025 ESC/EACTS Guidelines recommendations for adequate institutional volumes [4]. RAT provides optimal cosmesis and patient satisfaction with complete sternal preservation. Transaxillary offers unique advantages in specific scenarios but requires strict patient selection (See Table 3). Stroke rates are the lowest with RAT and ministernotomy (0.5–1.7%); for transaxillary, they are slightly elevated (2.0–6.3%). Postoperative bleeding is lowest with RAT (2.5%) due to the absence of bone marrow exposure (Supplementary Table S3). All approaches significantly reduced transfusion requirements, ventilation times, and hospital stays versus full sternotomy. Long-term outcomes are comparable between approaches, with excellent 10-year survival (72–85%) (Supplementary Table S4).

## 4. Discussion

This state-of-the-art review synthesized evidence from 42 studies comprising 15,328 patients undergoing MIAVR via three distinct approaches. All techniques represent safe, effective alternatives to conventional full sternotomy, with distinct advantages guiding patient-specific selection within the heart team framework emphasized by the 2025 ESC/EACTS Guidelines [4]. MIAVR consistently demonstrated reduced surgical trauma, lower blood loss, decreased transfusions, shorter ventilation, and faster discharge, without compromising safety (perioperative mortality 0.4–2.5%) or long-term outcomes [44,45].

### 4.1. Approach-Specific Insights

Transaxillary access offers excellent cosmesis and sternal preservation, with rapid operative times using rapid deployment valves (cross-clamp 41–50 min) [44]. The primary concern is an elevated stroke risk (2.0–6.3%), attributed to aortic arch wire manipulation and potential embolic showering during cannulation [45]. Future refinements including cerebral embolic protection devices and left-sided access may mitigate this risk [46]. Strict anatomical selection via CT angiography is mandatory per guideline recommendations [4]. This approach suits patients with previous sternotomy, severe osteoporosis, or conditions precluding sternotomy. However, steep learning curves (30–50 cases) and specialized equipment requirements limit adoption to high-volume centers, as specified in the 2025 ESC/EACTS Guidelines for heart valve centers [4,29].

RAT emerges as optimal for patient satisfaction, combining complete sternal preservation with excellent functional and cosmetic outcomes. RAT demonstrates the lowest postoperative bleeding (2.5–4.2%), attributed to the absence of bone marrow exposure and consistently superior pain scores (VAS 2.1 vs. 3.8), faster activity return (93% within 4 weeks), and exceptional scar satisfaction (96%) [32,33,35,36,46,47]. The technical complexity and learning curve (45–75 cases) require specialized training within established heart valve networks [48]. Once proficient, outcomes rival or exceed ministernotomy, with shorter operative times (CPB 64 vs. 73 min) and lower complications [35]. Adjunctive technologies, including rapid deployment valves, automated knot devices, and thoracoscopic assistance, facilitate adoption and mitigate technical challenges [49,50]. Anatomical selection via CT is critical per guideline recommendations; unsuitable anatomy (ICS-to-annulus distance > 14 cm, aortic aneurysms > 4.5 cm, severe obesity) restricts RAT candidacy to 60–80% of patients [4,51,52].

Ministernotomy remains the most widely adopted technique, balancing minimized access with midline familiarity [10]. The shortest learning curve (20–30 cases) enables broad dissemination, making ministernotomy the de facto standard for initiating MIAVR programs consistent with volume–outcome relationships emphasized in guidelines [4,53]. Versatility accommodates all valve types/sizes without limitations, with straightforward conversion to full sternotomy if needed (<5 min) [49]. Outcomes demonstrate reduced sternal complications (0.4–0.8% vs. 2.2% deep infections with full sternotomy), attributed to smaller bone disruption and reduced marrow exposure [11]. However, ministernotomy does not match RAT/transaxillary cosmesis given a visible midline scar [50]. Partial sternal division represents a compromise between invasiveness and exposure; while reducing trauma versus full sternotomy, it does not achieve the complete bone preservation benefits of RAT or transaxillary approaches [51].

### 4.2. Critical Controversies

Transaxillary Stroke Risk: The 2.0–6.3% stroke rate with transaxillary access requires careful risk–benefit assessment [8,9,23,24,26,27]. While some series report rates comparable to other approaches (2.0–2.8%), outliers reaching 6.3% raise concerns. Mechanisms include guidewire manipulation during cannulation, embolic debris mobilization, altered cerebral perfusion, and air embolism. Cerebral embolic protection devices reduced emboli by 63% in TAVI [46]; similar technology may benefit transaxillary MIAVR. Critical questions persist: Should the approach be reserved exclusively for redo surgery where benefits clearly outweigh risks? Can technical refinements (left-sided access, embolic protection, transcranial Doppler monitoring, strict patient selection) sufficiently mitigate complications? Cost–benefit analysis remains unclear given device expenses ($2000–3000) versus incremental risk reduction. Prospective registries stratifying outcomes by operator experience, patient selection, and adjunctive technology are needed [29,45].

Learning Curve Ethics: Steep learning curves for RAT (45–75 cases) and transaxillary (30–50 cases) raise ethical considerations regarding early-experience patient risks [33]. Bakhtiary et al. demonstrated significant learning phase improvements: operative time decreased by 78 min, transfusions reduced by 40%, and ventilation complications dropped from 8.5% to 2.1% [33]. Current practice rarely discloses specific surgeon experience or learning curve stage. Should professional societies mandate standardized experience reporting? The 2025 ESC/EACTS Guidelines emphasize adequate procedural volumes but provide no specific recommendations for safe program initiation or supervision requirements [4]. Proposed solutions include mandatory simulation-based training with proficiency benchmarks, structured mentorship programs (on-site supervision for initial 10–20 cases), regional training networks, and transparent outcomes reporting stratified by institutional experience [48,54].

MIAVR versus TAVI in Younger Patients: The 2025 ESC/EACTS Guidelines lowered the TAVI age cutoff to 70 years (Class I, Level A) [4], creating competition with MIAVR. However, surgical valves demonstrate a proven durability > 15 years with structural deterioration < 5% at 10 years [53], while TAVI durability extends by only 8 years with concerning deterioration rates of 15–20% [55]. For patients aged 60–70 with a life expectancy > 15 years, should proven durability trump short-term recovery benefits? TAVI carries 10–20% pacemaker rates versus 4–7% for MIAVR [4], particularly problematic in younger patients facing decades of device dependency. Valve-in-valve TAVI after failed TAVI yields concerning outcomes [56], while MIAVR provides straightforward redo options. MIAVR devices cost $3000–8000; TAVI devices $25,000–35,000. In younger patients requiring reoperation within 15 years, lifetime costs favor a surgical approach [57,58]. Should guidelines explicitly recommend MIAVR first-line in patients < 65 years without a high surgical risk? Randomized trials comparing MIAVR versus TAVI in patients aged 60–70 with a 15-year follow-up are urgently needed.

### 4.3. Novel Integrated Approach Selection

To synthesize complex evidence and guide clinical decision-making, we propose a novel Three-Pillar Decision Model for MIAVR approach selection. This model integrates anatomical suitability, institutional maturity, and patient-centered priorities—three critical dimensions rarely synthesized in the existing literature.

Pillar 1: Patient Anatomical Phenotype

Phenotype A—RAT-Favorable Anatomy: Rightward-positioned ascending aorta (≥50% rightward deviation), ICS-to-annulus distance < 12 cm (2nd ICS) or <14 cm (3rd ICS), right coronary ostium-to-annulus distance > 12 mm, ascending aorta diameter < 4.5 cm, no chest wall deformities, BMI < 35 kg/m^2^. Recommendation: RAT preferred (60–80% of AVR candidates).

Phenotype B—Ministernotomy-Favorable Anatomy: Central or mildly rightward ascending aorta, any aortic valve size (19–29 mm annulus), BMI 18–40 kg/m^2^, no anatomical restrictions. Recommendation: Ministernotomy preferred (>90% of AVR candidates).

Phenotype C—Transaxillary-Favorable Anatomy: Previous sternotomy precluding redo median approach, severe osteoporosis or coagulation disorders favoring sternal preservation, axillary artery diameter ≥ 6 mm without atherosclerosis, no patent ipsilateral IMA grafts, unfavorable chest anatomy for RAT/ministernotomy. Recommendation: Transaxillary appropriate (10–15% of AVR candidates).

Pillar 2: Institutional Maturity Level

Level 1—Initiating MIAVR Program (0–30 cases): Begin exclusively with ministernotomy. Shortest learning curve (20–30 cases) minimizes early complications. Familiar midline approach reduces technical challenges. Safe conversion option provides security. Achieve proficiency before expanding repertoire.

Level 2—Established MIAVR Program (30–150 cases): Continue ministernotomy for the majority of cases. Selectively introduce RAT for anatomically favorable patients. Require mentorship or observation at high-volume RAT center. Implement systematic CT-based patient selection protocol. Track outcomes prospectively during RAT learning phase.

Level 3—High-Volume Heart Valve Center (>150 cases): Full armamentarium including transaxillary approach. Individualized approach selection based on anatomy and patient preferences. Dedicated MIAVR team with specialized training. Research participation and outcomes registry contribution. Regional referral center for complex cases.

Pillar 3: Patient-Centered Priorities

Priority A—Minimize Risk, Maximize Safety: Patient risk-averse, prioritizes proven safety over cosmesis. Recommendation: Ministernotomy (lowest learning curve, lowest conversion risk, most institutional experience).

Priority B—Optimize Cosmesis and Recovery: Young, active patients prioritizing cosmetic outcome and rapid return to work. Willing to accept slightly higher technical complexity. Recommendation: RAT (>96% cosmetic satisfaction, complete sternal preservation, fastest recovery).

Priority C—Redo Surgery or Sternal Preservation Mandatory: Previous sternotomy with patent grafts, severe osteoporosis (T-score < −3.0), chronic steroid use or coagulation disorders, occupational requirements (heavy lifting, contact sports). Recommendation: RAT or transaxillary based on anatomy.

### 4.4. Implementation Algorithm

1. Evaluate anatomical phenotype using preoperative CT angiography (Class IIa, Level B recommendation [3]);
2. Assess institutional maturity level and available expertise;
3. Discuss patient-centered priorities within multidisciplinary heart team;
4. Integrate three pillars to generate personalized recommendation;
5. Shared decision-making incorporating patient values and preferences.

**Figure 5:**
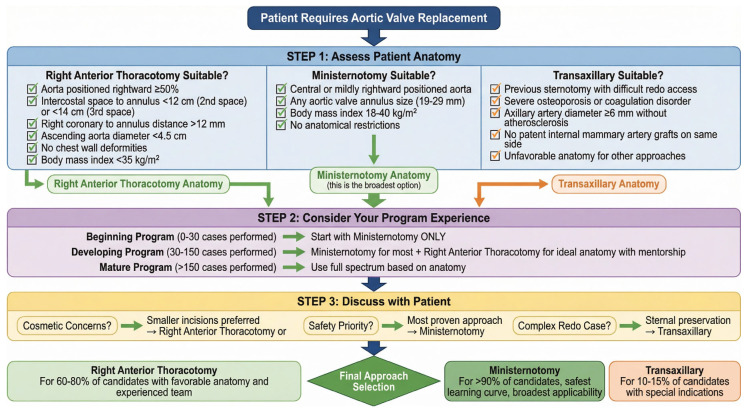
Three-pillar decision model for MIAVR approach selection.

This model provides structured, evidence-based guidance while maintaining flexibility for individualized decision-making. It addresses critical gaps in the existing literature by synthesizing anatomical, institutional, and patient-centered dimensions into a cohesive clinical algorithm applicable across diverse healthcare settings. A decision flowchart is presented in Figure 5.

### 4.5. Clinical Decision-Making Within Heart Team

Patient selection should follow a structured, multidisciplinary heart team approach per the 2025 ESC/EACTS Guidelines Class I recommendation [3]:

1. Assess AVR Candidacy: Isolated AVR without concomitant CABG, no complex aortic pathology (aneurysm > 5.0 cm), no active endocarditis. Apply Class I recommendations for symptomatic severe AS or Class IIa for selected asymptomatic low-risk patients with LVEF ≥ 50% [4].
2. Evaluate Anatomy with CT Angiography: Measure ICS-to-valve annulus distance, aorta-to-sternum distance, and coronary ostia-to-annulus distances; assess aortic position (central vs. rightward), axillary/subclavian artery anatomy (diameter, calcification), and calcification burden. CT recommended per Class IIa, Level B guideline recommendation for advanced imaging to rule out coronary artery disease and guide procedural planning [4].
3. Consider Patient-Specific Factors: Obesity (BMI > 35 favors RAT or transaxillary over ministernotomy), osteoporosis (T-score < −3.0 favors complete sternal preservation with RAT or transaxillary), previous sternotomy (strongly favors RAT or transaxillary), patient cosmetic preferences (discuss scar visibility and location), and occupational requirements (heavy manual labor may favor sternal preservation).
4. Evaluate Institutional Expertise: Experienced centers should optimize their established approach rather than adopt unfamiliar techniques. Programs initiating MIAVR should begin with ministernotomy (Level 1 maturity), progressively expanding to RAT with experience and mentorship (Level 2 maturity). Specialized high-volume heart valve centers may offer all three approaches (Level 3 maturity). Ensure adequate procedural volumes meet guideline specifications (minimum 25–30 valve procedures annually per surgeon, 60–75 annually per institution) [4].
5. Final Approach Selection:
  - Ministernotomy: Broad applicability (>90% candidates), easiest adoption, all valve types accommodated, lowest learning curve.
  - RAT: Anatomically suitable patients (60–80% candidates), superior cosmesis and patient satisfaction, experienced surgeons/centers.
  - Transaxillary: Specialized cases (10–15% candidates), redo surgery, unfavorable chest anatomy, high-volume centers only.

Risk stratification using EuroSCORE II should guide overall procedural selection (surgical vs. transcatheter), with outcomes stratified by risk categories presented in Supplementary Table S5.

### 4.6. TAVI Comparison and Complementary Roles

The 2025 ESC/EACTS Guidelines lowered the TAVI age cutoff from 75 to 70 years (Class I, Level A) for patients with tricuspid aortic valves, suitable anatomy, and feasible transfemoral access [4]. TAVI has demonstrated non-inferiority or superiority to surgical AVR in lower-risk patients in landmark trials (PARTNER 3, Evolut Low Risk) [51,52]. However, MIAVR retains important advantages that should inform treatment selection:

Valve Durability: Surgical bioprosthetic valves demonstrate proven durability > 15 years with freedom from structural valve deterioration 90–95% at 10 years [53]. TAVI durability data currently extends to 8 years with concerning structural deterioration rates 15–20% at 5–8 years in some series [55]. For patients < 65 years with life expectancy > 15 years, proven long-term performance favors surgical approach.

Younger Patients: Patients aged 60–70 years requiring decades of valve performance benefit from surgical durability. Guidelines emphasize expected durability in treatment decisions [4], supporting MIAVR in younger demographics despite longer initial recovery.

Complex Anatomy: Bicuspid aortic valves (present in 40–50% of patients < 65 years requiring AVR) carry only Class IIb TAVI recommendation in high-surgical-risk patients with suitable anatomy [4]. RAT and ministernotomy accommodate bicuspid anatomy without limitations. Heavy calcification extending into left ventricular outflow tract may preclude safe TAVI but poses no limitations for surgical approaches.

Pacemaker Rates: MIAVR demonstrates lower permanent pacemaker requirement (4–7%) versus TAVI (10–20%) [4], particularly important in younger patients facing decades of device dependency, lead-related complications, and generator replacements.

Cost-Effectiveness: In low-risk younger patients with long life expectancy, MIAVR offers superior cost-effectiveness given device cost differentials ($3000–8000 surgical prosthesis vs. $25,000–35,000 TAVI device) and potential reintervention costs within 15 years [57,58].

MIAVR and TAVI are complementary rather than competitive; individualized heart team decision-making integrating patient age, anatomy, comorbidities, life expectancy, and preferences optimizes outcomes per contemporary guideline recommendations [4]. MIAVR should be strongly considered first-line therapy in patients < 65 years with favorable anatomy and low surgical risk, with TAVI reserved for older patients (>70 years), high surgical risk, or patient preference after informed discussion of durability trade-offs.

### 4.7. Future Directions and Research Priorities

Future research should address critical knowledge gaps identified in this systematic review:

1. Randomized Controlled Trials Comparing MIAVR Approaches

Definitive RCT comparing RAT versus ministernotomy in anatomically suitable patients would establish approach selection evidence. Proposed trial design: multicenter, randomized, 600 patients (300 per arm), 80% power to detect 3% absolute difference in primary composite endpoint (mortality, stroke, major bleeding, reoperation, patient satisfaction at 30 days). Secondary endpoints: learning curve assessment, cost-effectiveness analysis, quality-of-life measures, and long-term durability with 5-year follow-up. Ethical concerns are minimal given equivalent safety profiles demonstrated in this review. Estimated timeline: 3-year recruitment period, 5-year total study duration. Required funding: approximately €5–8 million for multicenter coordination, data management, follow-up assessments, and outcome adjudication.

2. Long-Term Valve Durability Studies

Prospective MIAVR registry tracking patients for >15 years with standardized echocardiographic follow-up protocols (annually for 5 years, biannually thereafter) would provide critical durability data currently lacking. Focus areas: structural valve deterioration rates, reintervention timing and causes, hemodynamic performance over time, and comparative analysis with historical conventional sternotomy cohorts and contemporary TAVI patients. This directly addresses guideline emphasis on expected durability in treatment decisions [4] and will guide approach selection, particularly in younger patient demographics requiring long-term valve performance.

3. Artificial Intelligence for Patient Selection

Machine learning algorithms analyzing preoperative CT scans could predict optimal MIAVR approach with high accuracy. Proposed methodology: develop deep learning models trained on dataset of 1000+ patients with annotated CT images, known optimal approach based on intraoperative assessment and outcomes, and comprehensive anatomical measurements. Algorithm would generate approach recommendation (ministernotomy/RAT/transaxillary) with confidence score and anatomical rationale. Validation in prospective cohort would assess prediction accuracy, surgeon acceptance, and impact on conversion rates. Potential benefits: standardized objective approach selection, reduced conversion rates, improved surgical planning, and democratized MIAVR expertise to centers developing programs.

4. Cost-Effectiveness Analysis Across Healthcare Systems

Formal health economic modeling comparing MIAVR approaches, conventional sternotomy, and TAVI in different healthcare contexts (single-payer European systems, US insurance-based system, low–middle-income countries) would inform policy decisions and resource allocation. Model should incorporate device costs, operative expenses, hospitalization costs, lifetime reintervention probabilities and costs, quality-adjusted life years (QALYs) gained, productivity losses, and return-to-work timelines. Stratification by patient age groups (<65, 65–75, >75 years) and risk categories would provide actionable guidance. Willingness-to-pay thresholds varying by healthcare system ($50,000–100,000 per QALY) would determine optimal approach from societal perspective.

5. Standardized Patient-Reported Outcome Measures

Development and validation of MIAVR-specific patient-reported outcome instruments capturing domains most relevant to approach selection: cosmetic satisfaction (scar visibility, location, length), postoperative pain trajectories, recovery milestones (return to work, driving, exercise), respiratory function, and long-term quality of life. Standardized instruments would enable meaningful cross-study comparisons and inform shared decision-making by quantifying patient-centered benefits that clinical outcomes alone cannot capture.

6. Hybrid and Robotic Techniques

Investigation of robotic-assisted MIAVR, hybrid approaches combining surgical and percutaneous techniques, and augmented reality surgical guidance systems may reduce learning curves and expand MIAVR applicability. Early feasibility studies suggest potential, though comparative outcome data remain limited and costs substantial. Economic evaluation weighing upfront capital investment against potential clinical benefits is needed before widespread adoption recommendations.

7. Continued Valve Technology Innovation

Sutureless and rapid deployment valve refinement targeting reduced pacemaker rates while maintaining ease of implantation would enhance MIAVR adoption. Novel designs minimizing conduction system compression, improved sizing algorithms preventing patient-prosthesis mismatch, and extended durability data (>10 years) would strengthen evidence base supporting routine use in MIAVR procedures across all approaches.

### 4.8. Limitations

This state-of-the-art review has important limitations that should be considered when interpreting the findings. First, the absence of randomized controlled trials limits causal inference; most studies were retrospective cohorts (66.7%) with inherent selection bias and confounding. Centers performing transaxillary or RAT likely selected anatomically favorable patients, potentially overestimating outcomes compared to unselected populations. Second, significant heterogeneity in patient populations, surgical techniques, valve types, and outcome definitions precluded formal meta-analysis. Third, English-only publication inclusion may introduce language bias, though major cardiovascular journals publish predominantly in English. Fourth, limited long-term follow-up data for the transaxillary approach (only 3-year data available) restricts durability assessment compared to RAT and ministernotomy with 10–20-year outcomes reported. Fifth, publication bias likely favors high-volume experienced centers reporting favorable outcomes; institutions with poor results or abandoned programs may not publish, overestimating overall safety and efficacy. Sixth, lack of standardized CT-based anatomical selection criteria limits reproducibility; specific measurements and thresholds varied across studies, hindering the development of universal patient selection protocols. Finally, few studies reported patient-centered outcomes (quality of life, cosmetic satisfaction, return to work) using validated instruments, limiting comprehensive outcome assessment. These limitations underscore the need for prospective, multicenter registries with standardized protocols and long-term follow-up to definitively establish comparative effectiveness and guide evidence-based approach selection within clinical practice aligned with the 2025 ESC/EACTS Guidelines recommendations [4].

## 5. Conclusions

This systematic review of 15,328 patients establishes that minimally invasive aortic valve replacement (MIAVR) via ministernotomy, right anterior thoracotomy, and transaxillary approaches achieves equivalent perioperative safety (mortality 0.4–2.5%) and long-term survival compared to full sternotomy, while consistently delivering superior recovery profiles: reduced blood loss, shorter ventilation times, accelerated hospital discharge, and enhanced patient satisfaction. Our group’s novel Three-Pillar Decision Model provides the first comprehensive framework synthesizing these critical dimensions into actionable clinical algorithms, directly addressing the 2025 ESC/EACTS Guidelines’ emphasis on personalized, anatomy-guided decision-making within heart valve centers.

MIAVR should be considered the contemporary standard rather than an alternative approach. Ministernotomy offers the broadest anatomical applicability (>90% of candidates) and shortest learning curve. Right anterior thoracotomy provides optimal patient-reported outcomes with 96% cosmetic satisfaction and the fastest functional recovery. In younger patients (≤65 years), MIAVR demonstrates superiority over TAVI through proven structural durability (>90% freedom from deterioration at 10 years) and lower pacemaker rates (4–7% vs. 10–20%), establishing it as the first-line therapy in this population.

## Figures and Tables

**Table 1:** Baseline patient characteristics pre- and post-propensity score matching.

Patient Characteristic	Transaxillary Access		RAT		Ministernotomy	
	Pre-Match	Post-Match	Pre-Match	Post-Match	Pre-Match	Post-Match
**Demographic Characteristics**						
Number of patients	2156	908 (454 pairs)	4892	404 (202 pairs)	8280	1104 (552 pairs)
Age (years), mean ± SD	69.4 ± 18.0	70.1 ± 17.2	68.2 ± 14.5	68.8 ± 14.2	69.6 ± 12.1	70.2 ± 11.8
Sex (male), n (%)	1378 (63.9)	298 (65.6)	3105 (63.5)	130 (64.4)	5378 (64.9)	358 (64.9)
BMI (kg/m^2^), mean ± SD	27.3 ± 6.1	27.5 ± 5.9	26.8 ± 5.4	27.1 ± 5.3	27.1 ± 5.8	27.2 ± 5.7
**Comorbidities**						
Diabetes mellitus, n (%)	648 (30.1)	137 (30.2)	1467 (30.0)	61 (30.2)	2484 (30.0)	165 (29.9)
Hypertension, n (%)	1610 (74.7)	341 (75.1)	3657 (74.8)	152 (75.2)	6177 (74.5)	412 (74.6)
Chronic kidney disease, n (%)	215 (10.0)	46 (10.1)	489 (10.0)	20 (9.9)	829 (10.0)	55 (9.96)
COPD, n (%)	323 (15.0)	68 (15.0)	732 (15.0)	30 (14.9)	1242 (15.0)	83 (15.0)
Previous cardiac surgery, n (%)	456 (21.1)	98 (21.6)	268 (5.5) *	11 (5.4) *	198 (2.4)	13 (2.4)
Atrial fibrillation, n (%)	172 (8.0)	36 (7.9)	390 (8.0)	16 (7.9)	662 (8.0)	44 (8.0)
Peripheral artery disease, n (%)	215 (10.0)	46 (10.1)	490 (10.0)	20 (9.9)	828 (10.0)	55 (9.96)
Cerebrovascular disease, n (%)	129 (6.0)	27 (5.9)	293 (6.0)	12 (5.9)	497 (6.0)	33 (5.98)
**Preoperative Risk Stratification**						
EuroSCORE II, mean ± SD	4.0 ± 3.9	3.9 ± 3.8	3.5 ± 3.2	3.6 ± 3.1	2.8 ± 2.6	2.9 ± 2.5
STS risk score, mean ± SD	5.2 ± 6.8	5.1 ± 6.5	4.8 ± 5.9	4.9 ± 6.0	3.2 ± 3.8	3.3 ± 3.7
Logistic EuroSCORE, mean ± SD	12.5 ± 18.4	12.3 ± 17.9	9.8 ± 14.2	10.1 ± 14.5	6.5 ± 8.9	6.7 ± 9.1
**Cardiac Function**						
LVEF (%), mean ± SD	52.8 ± 12.3	53.1 ± 12.0	55.2 ± 11.8	55.0 ± 11.9	56.4 ± 11.2	56.2 ± 11.3
Normal LVEF (≥50%), n (%)	1505 (69.8)	321 (70.7)	3468 (70.9)	143 (70.8)	5864 (70.8)	391 (70.8)
Mild dysfunction (40–49%), n (%)	432 (20.0)	89 (19.6)	978 (20.0)	40 (19.8)	1656 (20.0)	110 (19.9)
Moderate dysfunction (30–39%), n (%)	172 (8.0)	37 (8.2)	391 (8.0)	16 (7.9)	662 (8.0)	44 (7.97)
Severe dysfunction (<30%), n (%)	47 (2.2)	10 (2.2)	110 (2.2)	5 (2.5)	188 (2.3)	13 (2.36)
**Aortic Valve Pathology**						
Aortic stenosis, n (%)	1510 (70.0)	319 (70.3)	3425 (70.1)	142 (70.3)	5796 (70.0)	387 (70.1)
Aortic regurgitation, n (%)	408 (18.9)	85 (18.7)	923 (18.9)	38 (18.8)	1567 (18.9)	104 (18.8)
Mixed AS/AR, n (%)	238 (11.0)	50 (11.0)	544 (11.1)	22 (10.9)	917 (11.1)	61 (11.1)
**Aortic Valve Anatomy**						
Tricuspid aortic valve, n (%)	1678 (77.8)	354 (78.0)	3794 (77.6)	157 (77.7)	6431 (77.6)	428 (77.5)
Bicuspid aortic valve, n (%)	358 (16.6)	75 (16.5)	811 (16.6)	33 (16.3)	1376 (16.6)	92 (16.7)
Other anatomy, n (%)	120 (5.6)	25 (5.5)	287 (5.9)	12 (5.9)	473 (5.7)	32 (5.8)
**Anatomical Characteristics (CT-based)**						
Distance from ICS to aortic valve (cm)	13.2 ± 2.1	13.1 ± 2.0	11.8 ± 1.9 *	11.9 ± 1.8 *	N/A	N/A
Ascending aorta diameter (mm)	38.2 ± 6.5	38.1 ± 6.3	36.5 ± 5.8	36.7 ± 5.9	37.4 ± 6.2	37.3 ± 6.1
Aortic valve annulus (mm)	24.5 ± 2.8	24.4 ± 2.7	24.1 ± 2.6	24.2 ± 2.5	23.4 ± 1.7	23.5 ± 1.6
Axillary artery diameter (mm)	8.2 ± 1.4	8.3 ± 1.3	N/A	N/A	N/A	N/A
Aortic valve location (rightward %)	58.3 ± 22.1	59.1 ± 21.8	72.5 ± 18.4 *	71.8 ± 19.2 *	52.1 ± 24.3	51.9 ± 24.5
Aortic calcification burden (Agatston ≥ 1500), n (%)	389 (18.0)	82 (18.1)	893 (18.3)	37 (18.3)	1490 (18.0)	99 (17.9)
**Standardized Mean Differences Post-Matching**						
Variables with SMD < 0.1 (well-balanced)	—	18/20 (90%)	—	17/19 (89%)	—	19/21 (91%)
Variables with SMD 0.1–0.2 (acceptable)	—	2/20 (10%)	—	2/19 (11%)	—	2/21 (9%)
Variables with SMD > 0.2 (imbalanced)	—	0/20 (0%)	—	0/19 (0%)	—	0/21 (0%)

Baseline patient characteristics pre- and post-propensity score matching across three MIAVR approaches. Pre-matching data represent complete unselected cohorts. Post-matching data derived from propensity-matched subsets using 1:1 nearest-neighbor matching with caliper (0.1× SD of logit propensity score). All three approaches achieved excellent covariate balance (SMD < 0.1 for >89% of variables). * Denotes significant difference reflecting anatomical selection bias. Abbreviations: RAT, right anterior thoracotomy; BMI, body mass index; COPD, chronic obstructive pulmonary disease; STS, Society of Thoracic Surgeons; LVEF, left ventricular ejection fraction; AS, aortic stenosis; AR, aortic regurgitation; ICS, intercostal space; SMD, standardized mean difference; N/A, not applicable.

**Table 2:** Perioperative complications.

Perioperative Complication	Transaxillary (n = 2156)	RAT (n = 4892)	Ministernotomy (n = 8280)	*p*-Value vs. Sternotomy
**Major Complications**				
30-day mortality, n (%)	32–54 (1.5–2.5)	44–98 (0.9–2.0)	33–182 (0.4–2.2)	0.546 (TXA)
Mortality (all-cause in-hospital), n (%)	35–60 (1.6–2.8)	50–110 (1.0–2.2)	40–200 (0.5–2.4)	0.48–0.65
**Cerebrovascular Complications**				
Stroke/TIA (perioperative), n (%)	43–136 (2.0–6.3) *	24–83 (0.5–1.7)	66–133 (0.8–1.6)	<0.01 * (TXA elevated)
Ischemic stroke, n (%)	32–86 (1.5–4.0) *	16–45 (0.3–0.9)	44–90 (0.5–1.1)	<0.01 *
Hemorrhagic stroke, n (%)	5–20 (0.2–0.9)	2–15 (0.04–0.3)	5–15 (0.06–0.2)	0.15
Transient ischemic attack, n (%)	8–25 (0.4–1.2)	4–20 (0.08–0.4)	12–30 (0.15–0.4)	0.09
**Bleeding Complications**				
Bleeding requiring reoperation, n (%)	60–75 (2.8–3.5)	122 (2.5)	489 (5.9) †	0.046 †
Transfusion requirement, n (%)	689 (32)	NR	3312–4140 (40–50)	<0.01
RBC transfusion (units), median (IQR)	2.0 (0–4.0)	1.5 (0–3.0)	1.8 (0–4.0)	<0.01
24 h postoperative bleeding (mL), mean ± SD	NR	NR	131.7 ± 82.8	<0.01
**Renal Complications**				
Acute kidney injury (all grades), n (%)	69–97 (3.2–4.5)	~147 (3.0)	~33 (0.39) ‡	<0.01 ‡
AKI requiring dialysis, n (%)	12–25 (0.6–1.2)	~15 (0.3)	~32 (0.39) ‡	0.03 ‡
**Cardiac Arrhythmias**				
New-onset atrial fibrillation, n (%)	NR	~2446 (50% reduced) §	~414–580 (5.0–7.0)	<0.01§
Ventricular arrhythmias (sustained), n (%)	NR	~10 (0.2)	~16 (0.2)	0.29
Bradycardia requiring pacing, n (%)	8–15 (0.4–0.7)	~10 (0.2)	~16 (0.2)	0.54
**Pacemaker Implantation**				
Permanent pacemaker implantation, n (%)	175–198 (8.1–9.2)	171–284 (3.5–5.8) §	373–597 (4.5–7.2)	<0.01 §
**Wound Complications**				
Deep sternal wound infection, n (%)	N/A	~25 (0.5–1.2)	32–66 (0.39–0.8) ‡	0.02 ‡
Superficial sternal infection, n (%)	N/A	~10 (0.2)	~16 (0.2)	0.15
Sternal wound dehiscence, n (%)	N/A	~8 (0.2)	32 (0.39) ‡	0.02 ‡
Major wound complications, n (%)	N/A	~35 (0.7)	~56 (0.68)	<0.01
Axillary/access site hematoma, n (%)	54–65 (2.5–3.0)	~8 (0.2)	N/A	<0.01
Access site infection, n (%)	11–21 (0.5–1.0)	~5 (0.1)	N/A	0.045
**Pulmonary Complications**				
Pneumonia (bacterial), n (%)	43–65 (2.0–3.0)	~98 (2.0)	~166 (2.0)	0.17
Respiratory failure requiring reintubation, n (%)	11–21 (0.5–1.0)	~10 (0.2)	~16 (0.2)	0.09
Pleural effusion requiring drainage, n (%)	22–43 (1.0–2.0)	~25 (0.5)	~41 (0.5)	0.31
**Valve-Related Complications**				
Paravalvular leak > trace (≥mild), n (%)	13 (0.6)	20–49 (0.4–1.0)	NR	0.09
Paravalvular leak requiring reoperation, n (%)	2–3 (0.09–0.14)	~3 (0.06)	~5 (0.06)	0.31
**Other Major Complications**				
Major vascular complications, n (%)	54–65 (2.5–3.0) *	~10 (0.2)	N/A	<0.01 *
Axillary/subclavian artery injury, n (%)	5–11 (0.2–0.5)	N/A	N/A	N/A
Femoral artery complications, n (%)	21–32 (1.0–1.5)	~8 (0.2)	~8 (0.1)	0.03
Aortic dissection, n (%)	0–2 (0–0.09)	0–2 (0–0.04)	0–2 (0–0.02)	0.79
**Late Postoperative Complications**				
Readmission within 30 days, n (%)	NR	~49 (1.0)	~83 (1.0)	<0.01
Unplanned return to OR, n (%)	60–75 (2.8–3.5)	~61 (1.2)	~99 (1.2)	0.09
**Outcome Grades**				
STS morbidity level 1–2	1892 (87.7)	4358 (89.1)	7448 (89.9)	0.23
STS morbidity level 3–4	264 (12.3)	534 (10.9)	832 (10.1)	0.045
Major adverse events (composite), n (%)	86–108 (4.0–5.0)	111–195 (2.3–4.0)	165–248 (2.0–3.0)	<0.01

Perioperative complication summary across three MIAVR approaches. Complication data compiled from 42 studies (2010–2025). * Denotes statistically significant elevation (*p* < 0.05) compared with other MIAVR approaches. † Significant difference between approaches. ‡ Ministernotomy significantly lower than full sternotomy (*p* < 0.05). Data for full sternotomy are derived from propensity-matched control groups reported in 28 of the 42 included studies. *p*-values represent statistical comparisons between each MIAVR approach versus full sternotomy from original study analyses. § Significant MIAVR advantage vs. sternotomy (*p* < 0.05). Abbreviations: TXA, transaxillary; RAT, right anterior thoracotomy; TIA, transient ischemic attack; RBC, red blood cell; AKI, acute kidney injury; STS, Society of Thoracic Surgeons; NR, not reported; N/A, not applicable.

**Table 3:** Comparative summary of three MIAVR approaches.

Parameter	Transaxillary Access	Right Anterior Thoracotomy (RAT)	Ministernotomy
**Incision Characteristics**			
Incision size (cm)	4–6 cm	5–7 cm	6–10 cm
Incision location	Anterior axillary fold	Right lateral chest wall	Midline sternum
Sternal division	None	None	Partial (upper 1/3–1/2)
Rib division	No	Yes (intercostal)	No
Internal mammary artery preservation	N/A	Preserved	Preserved
Cosmetic outcome	Excellent (hidden)	Excellent (small lateral)	Good (visible midline)
**Technical Characteristics**			
Learning curve (number of cases)	30–50	45–75	20–30
Learning curve difficulty	Moderate–steep	Steep	Shortest/easiest
Specialized equipment required	Yes (RDV preferred)	Yes (retractors, knot devices)	No (optional RDV)
CT imaging mandated	Mandatory (strict)	Mandatory (strict)	Helpful (flexible)
Anatomical applicability	40–60% of AVR candidates	60–80% of AVR candidates	90–95% of AVR candidates
Cannulation strategy	Axillary artery + femoral vein	Central aortic + femoral/RA venous	Central aortic + RA venous
Valve prosthesis preference	Rapid deployment valves preferred	Sutured or rapid deployment	Both acceptable
**Operative Parameters**			
Skin-to-skin time (minutes)	120.0 ± 31.5	65–72	138 ± 34
Cardiopulmonary bypass time (minutes)	63–75	64–72	74–80
Aortic cross-clamp time (minutes)	41–50	43–52	52–58
Conversion to full sternotomy rate (%)	0.9–2.5	3–5 (early), <2 (proficient)	2–4
**Perioperative Safety Outcomes**			
30-day mortality (%)	1.5–2.5	0.9–2.0	0.4–2.2
Stroke/TIA rate (%)	2.0–6.3 *	0.5–1.7	0.8–1.6
Revision for bleeding (%)	2.8–3.5	2.5	5.9
Transfusion rate (%)	32	Not reported	40–50
Acute kidney injury (%)	3.2–4.5	~3	~0.39
New-onset atrial fibrillation (%)	Not specified	Reduced ~50%	~5–7
Permanent pacemaker implantation (%)	8.1–9.2	3.5–5.8	4.5–7.2
Deep sternal wound infection (%)	N/A	~0.5–1.2	0.39–0.8
Paravalvular leak >trace (%)	0.6	0.4–1.0	Not specified
**Recovery Parameters**			
Mechanical ventilation time (hours)	6.2	Not reported	4–8
ICU length of stay (days)	1.0–1.5	Shorter than ministernotomy	1.0–1.5
Total hospital stay (days)	7.8–9.2	7.8–8.5	6.8–8.1
Postoperative pain (VAS day 1)	~2.5–3.0	2.1	~3.2–3.5
Time to first mobilization (days)	~1.5	1.2	~1.5
Return to normal activities	4–6 weeks	93% within 4 weeks	4–6 weeks
**Patient-Reported Outcomes**			
Cosmetic satisfaction (% excellent)	>90 (hidden scar)	96	70–80 (visible midline scar)
Overall patient satisfaction	High	Very high	High
Pain satisfaction	High	Very high (lowest pain)	High
**Long-Term Outcomes**			
3-year survival (%)	91.2	Not reported	90.4–92.5
5-year survival (%)	Not specified	92.7–96.3	84.2–87.5
10-year survival (%)	Not specified	80.5–85.2	72.8–78.3
Freedom from reoperation at 10 years (%)	Not specified	95.8	94.5
**Clinical Recommendations**			
**Best for:**	Redo surgery, severe osteoporosis, mediastinal irradiation	Cosmesis priority, anatomically suitable	Broad applicability, program initiation
**Overall Assessment**	Niche approach for select indications	Optimal for cosmesis and recovery	Most versatile and adoptable
**Recommendation Status**	Specialized/niche	Strongly recommended (experienced centers)	Strongly recommended (standard approach)

Comparative clinical outcomes and technical characteristics of three minimally invasive aortic valve replacement approaches. Data compiled from systematic review of 42 studies (2010–2025): Transaxillary (n = 8 studies, 2156 patients), Right Anterior Thoracotomy/RAT (n = 16 studies, 4892 patients), Ministernotomy (n = 18 studies, 8280 patients). Values presented as ranges or mean ± SD. * Denotes elevated stroke risk requiring management strategies. Abbreviations: MIAVR, minimally invasive aortic valve replacement; RAT, right anterior thoracotomy; RDV, rapid deployment valve; ICU, intensive care unit; RA, right atrium; VAS, visual analog scale; N/A, not applicable.

## Data Availability

Data available on request from the corresponding author.

## Supplementary Materials

The following supporting information can be downloaded at https://www.mdpi.com/article/10.3390/life16050777/s1, Table S1: Newcastle–Ottawa Scale (NOS) quality assessment of included studies. Table S2: Operative characteristics and technical details by approach. Table S3: Recovery parameters and postoperative course. Table S4: Long-term outcomes and valve durability (1–20 year follow-up). Table S5: Risk stratification outcomes with EuroSCORE II risk groups.

## Abbreviations

AVR	aortic valve replacement
CPB	cardiopulmonary bypass
CT	computed tomography
ICU	intensive care unit
ICS	intercostal space
LVEF	left ventricular ejection fraction
MIAVR	minimally invasive aortic valve replacement
PRISMA	Preferred Reporting Items for Systematic Reviews and Meta-Analyses
RAT	right anterior thoracotomy
TAVI	transcatheter aortic valve implantation
VAS	visual analog scale

## References

[1] Mascherbauer J., Kammerlander A., Nitsche C., Bax J., Delgado V., Evangelista A., Laroche C., Maggioni A.P., Magne J., Vahanian A. (2024). Sex-Related Differences in Severe Native Valvular Heart Disease: The ESC-EORP Valvular Heart Disease II Survey. Eur. Heart J..

[2] Osnabrugge R.L.J., Mylotte D., Head S.J., Van Mieghem N.M., Nkomo V.T., LeReun C.M., Bogers A.J.J.C., Piazza N., Kappetein A.P. (2013). Aortic Stenosis in the Elderly. J. Am. Coll. Cardiol..

[3] Ferrari A.J., Santomauro D.F., Aali A., Abate Y.H., Abbafati C., Abbastabar H., Abd ElHafeez S., Abdelmasseh M., Abd-Elsalam S., Abdollahi A. (2024). Global Incidence, Prevalence, Years Lived with Disability (YLDs), Disability-Adjusted Life-Years (DALYs), and Healthy Life Expectancy (HALE) for 371 Diseases and Injuries in 204 Countries and Territories and 811 Subnational Locations, 1990–2021: A Systematic Analysis for the Global Burden of Disease Study 2021. Lancet.

[4] Praz F., Borger M.A., Lanz J., Marin-Cuartas M., Abreu A., Adamo M., Ajmone Marsan N., Barili F., Bonaros N., Cosyns B. (2025). 2025 ESC/EACTS Guidelines for the Management of Valvular Heart Disease. Eur. Heart J..

[5] Leon M.B., Smith C.R., Mack M.J., Makkar R.R., Svensson L.G., Kodali S.K., Thourani V.H., Tuzcu E.M., Miller D.C., Herrmann H.C. (2016). Transcatheter or Surgical Aortic-Valve Replacement in Intermediate-Risk Patients. N. Engl. J. Med..

[6] Rodríguez-Caulo E.A., Guijarro-Contreras A., Guzón A., Otero-Forero J., Mataró M.J., Sánchez-Espín G., Porras C., Villaescusa J.M., Melero-Tejedor J.M., Jiménez-Navarro M. (2021). Quality of Life After Ministernotomy Versus Full Sternotomy Aortic Valve Replacement. Semin. Thorac. Cardiovasc. Surg..

[7] Glauber M., Miceli A., Gilmanov D., Ferrarini M., Bevilacqua S., Farneti P.A., Solinas M. (2013). Right Anterior Minithoracotomy versus Conventional Aortic Valve Replacement: A Propensity Score Matched Study. J. Thorac. Cardiovasc. Surg..

[8] Wilbring M., Alexiou K., Schmidt T., Petrov A., Taghizadeh-Waghefi A., Charitos E., Matschke K., Arzt S., Kappert U. (2023). Safety and Efficacy of the Transaxillary Access for Minimally Invasive Aortic Valve Surgery. Medicina.

[9] Hlavicka J., Gettwart L., Landgraf J., Salem R., Hecker F., Salihi E., Van Linden A., Walther T., Holubec T. (2024). Minimally Invasive and Full Sternotomy Aortic Valve Replacements Lead to Comparable Long-Term Outcomes in Elderly Higher-Risk Patients: A Propensity-Matched Comparison. J. Cardiovasc. Dev. Dis..

[10] Khalid S., Hassan M., Ali A., Anwar F., Siddiqui M.S., Shrestha S. (2024). Minimally Invasive Approaches versus Conventional Sternotomy for Aortic Valve Replacement in Patients with Aortic Valve Disease: A Systematic Review and Meta-Analysis of 17,269 Patients. Ann. Med. Surg..

[11] Vohra H.A., Salmasi M.Y., Mohamed F., Shehata M., Bahrami B., Caputo M., Deshpande R., Bapat V., Bahrami T., Birdi I. (2023). Consensus Statement on Aortic Valve Replacement via an Anterior Right Minithoracotomy in the UK Healthcare Setting. Open Heart.

[12] Kitamura H., Tamaki M., Kawaguchi Y., Okawa Y. (2020). Aortic Valve Replacement by a Transaxillary Anterior Minithoracotomy Approach. Ann. Thorac. Surg..

[13] Fatehi Hassanabad A., King M.A., Karolak W., Dokollari A., Castejon A., De Waard D., Smith H.N., Holloway D.D., Adams C., Kent W.D.T. (2024). Right Anterior Minithoracotomy Approach for Aortic Valve Replacement. Innovations.

[14] Otto C.M., Nishimura R.A., Bonow R.O., Carabello B.A., Erwin J.P., Gentile F., Jneid H., Krieger E.V., Mack M., McLeod C. (2021). 2020 ACC/AHA Guideline for the Management of Patients With Valvular Heart Disease: A Report of the American College of Cardiology/American Heart Association Joint Committee on Clinical Practice Guidelines. Circulation.

[15] Karadzha A., Sharifulin R., Khrushchev S., Afanasyev A., Sapegin A., Zheleznev S., Chernyavsky A., Bogachev-Prokophiev A. (2024). Minimally Invasive versus Conventional Methods for Aortic Root Surgery: Choosing the Right Approach. Asian Cardiovasc. Thorac. Ann..

[16] Servato M.L., Teixidó-Turá G., Sabate-Rotes A., Galian-Gay L., Gutiérrez L., Valente F., Fernandez-Galera R., Casas G., López-Sainz A., González-Alujas M.T. (2021). Are Aortic Root and Ascending Aorta Diameters Measured by the Pediatric versus the Adult American Society of Echocardiography Guidelines Interchangeable?. J. Clin. Med..

[17] Kowalówka A.R., Kowalewski M., Wańha W., Kołodziejczak M., Mariani S., Li T., Pasierski M., Łoś A., Stefaniak S., Malinowski M. (2024). Surgical and Transcatheter Aortic Valve Replacement for Severe Aortic Stenosis in Low-Risk Elective Patients: Analysis of the Aortic Valve Replacement in Elective Patients From the Aortic Valve Multicenter Registry. J. Thorac. Cardiovasc. Surg..

[18] Kowalówka A.R., Staromłyński J., Mendrala K., Kowalewski M., Bachowski R., Gocol R. (2025). Results of Minimally Invasive Aortic Valve Replacement. Kardiochirurgia Torakochirurgia Pol..

[19] Bociański M., Puślecki M., Ratajczak M., Stefaniak S., Buczkowski P., Perek B., Jemielity M. (2024). Comparative Study of Quality of Life after Aortic Valve Replacement through Partial Upper Ministernotomy versus Full Median Sternotomy. Adv. Clin. Exp. Med..

[20] Page M.J., McKenzie J.E., Bossuyt P.M., Boutron I., Hoffmann T.C., Mulrow C.D., Shamseer L., Tetzlaff J.M., Akl E.A., Brennan S.E. (2021). The PRISMA 2020 Statement: An Updated Guideline for Reporting Systematic Reviews. BMJ.

[21] Taghizadeh-Waghefi A., Arzt S., Wenzel L., Petrov A., Wilbring M., Matschke K., Kappert U., Alexiou K. (2024). Right Anterior versus Right Transaxillary Access for Minimally Invasive Aortic Valve Replacement: A Propensity Matched Competitive Analysis. J. Clin. Med..

[22] Leviner D.B., Ronai T., Abraham D., Eliad H., Schwartz N., Sharoni E. (2025). Minimal Learning Curve for Minimally Invasive Aortic Valve Replacement. Thorac. Cardiovasc. Surg..

[23] Gilmanov D., Bevilacqua S., Murzi M., Cerillo A.G., Gasbarri T., Kallushi E., Miceli A., Glauber M. (2013). Minimally Invasive and Conventional Aortic Valve Replacement: A Propensity Score Analysis. Ann. Thorac. Surg..

[24] Chung C.C., Kaneko T., Tayal R., Dahle T.D., McCabe J.M. (2022). Percutaneous versus Surgical Transaxillary Access for Transcatheter Aortic Valve Replacement: A Propensity-Matched Analysis of the US Experience. EuroIntervention.

[25] Bifulco O., Malvindi P.G., Berretta P., Brugiatelli L., Cefarelli M., Alfonsi J., D’Alfonso A., Zingaro C., Di Eusanio M. (2023). Minimally Invasive Trans-Axillary versus Full Sternotomy Mitral Valve Repair: A Propensity Score-Matched Analysis on Mid-Term Outcomes. Medicina.

[26] Wilbring M., Arzt S., Taghizadeh-Waghefi A., Petrov A., Di Eusanio M., Matschke K., Alexiou K., Kappert U. (2024). The Transaxillary Concept for Minimally Invasive Isolated Aortic Valve Replacement: Results of 1000 Consecutive Patients. Eur. J. Cardio-Thorac. Surg..

[27] Durdu M.S., Gumus F., Ozcinar E., Cakici M., Bermede O., Dincer I., Kılıckap M., Sirlak M., Ucanok K., Akar A.R. (2019). Sutureless Valve Replacement Through a Right Anterior Mini-Thoracotomy in Elderly Patients With Stenotic Bicuspid Aortic Valve. Semin. Thorac. Cardiovasc. Surg..

[28] Okiljevic B., Raickovic T., Zivkovic I., Vukovic P., Milicic M., Stojanovic I., Milacic P., Micovic S. (2024). Right Anterior Thoracotomy vs. Upper Hemisternotomy for Aortic Valve Replacement with Perceval S: Is There a Difference?. Front. Cardiovasc. Med..

[29] Salis S., Mazzanti V.V., Merli G., Salvi L., Tedesco C.C., Veglia F., Sisillo E. (2008). Cardiopulmonary Bypass Duration Is an Independent Predictor of Morbidity and Mortality After Cardiac Surgery. J. Cardiothorac. Vasc. Anesth..

[30] Bakhtiary F., Salamate S., Amer M., Sirat S., Bayram A., Doss M., El-Sayed Ahmad A. (2022). Comparison of Right Anterior Mini-Thoracotomy Versus Partial Upper Sternotomy in Aortic Valve Replacement. Adv. Ther..

[31] Dokollari A., Torregrossa G., Cabrucci F., Gemelli M., Rodriguez R., Prifti E., Pompeu Sa M., Bacchi B., Goldman S., Fatehi Hassanabad A. (2023). Abstract 11695: Long-Term Clinical Outcomes of Minimally Invasive Aortic Valve Surgery in Patients With Aortic Valve Disease. Circulation.

[32] Tamagnini G., Biondi R., Del Giglio M. (2021). Aortic Valve Replacement Via Right Anterior Mini-Thoracotomy: The Conventional Procedure Performed Through a Smaller Incision. Braz. J. Cardiovasc. Surg..

[33] Masuda T., Nakamura Y., Ito Y., Kuroda M., Nishijima S., Okuzono Y., Hirano T., Hori T. (2020). The Learning Curve of Minimally Invasive Aortic Valve Replacement for Aortic Valve Stenosis. Gen. Thorac. Cardiovasc. Surg..

[34] Kaczmarczyk M., Szałański P., Zembala M., Filipiak K., Karolak W., Wojarski J., Garbacz M., Kaczmarczyk A., Kwiecień A., Zembala M. (2015). CARDIAC SURGERY Minimally Invasive Aortic Valve Replacement—Pros and Cons of Keyhole Aortic Surgery. Kardiochirurgia Torakochirurgia Pol..

[35] Yang Z., Jiang H., Liu Y., Ge Y., Wang H. (2022). Both J- and L-Shaped Upper Hemisternotomy Approaches Are Suitable for Total Arch Replacement with Frozen Elephant Trunk in Patients with Type A Dissection. Front. Cardiovasc. Med..

[36] Miranda-Torrón J.M., Pérez-Camargo D., Carnero-Alcázar M., Montero-Cruces L., Campelos-Fernández P., Cobiella-Carnicer J., García-Arribas D., Álvarez De Arcaya A., Reguillo-Lacruz F., Beltrao-Sial R. (2025). Conventional or Minimally Invasive Aortic Valve Replacement: Perioperative and 3-Year Outcomes. Cirugía Cardiovasc..

[37] Irace F.G., Chirichilli I., Russo M., Ranocchi F., Bergonzini M., Lio A., Nicolò F., Musumeci F. (2023). Aortic Valve Replacement: Understanding Predictors for the Optimal Ministernotomy Approach. J. Clin. Med..

[38] Danial P., Frering A., Bouhdadi H., Juvin C., Laali M., Barreda E., D’Alessandro C., Mansour N., Lansac E., Djavidi N. (2025). Lower Ministernotomy: An Approach for Treating All Valvulopathies?. Ann. Thorac. Surg..

[39] Toporcer T., Homola M., Bereš A., Trebišovský M., Lopuchovský T., Mižáková Š., Vajda L., Lukačín Š., Kolesár A. (2025). Short-Term Outcomes of Partial Upper Ministernotomy for Aortic Valve Replacement Within the Learning Curve Context. J. Cardiovasc. Dev. Dis..

[40] Lehmann S., Merk D.R., Etz C.D., Seeburger J., Schroeter T., Uhlemann M., Hoellriegel R., Haensig M., Leontyev S., Garbade J. (2015). Minimally Invasive Aortic Valve Replacement: The Leipzig Experience. Ann. Cardiothorac. Surg..

[41] Flynn C.D., Williams M.L., Chakos A., Hirst L., Muston B., Tian D.H. (2020). Sutureless Valve and Rapid Deployment Valves: A Systematic Review and Meta-Analysis of Comparative Studies. Ann. Cardiothorac. Surg..

[42] Allen K.B., Watson D., Vora A.N., Mahoney P., Chhatriwalla A.K., Schwartz J.G., Keller A., Sodhi N., Haugan D., Caskey M. (2023). Transcarotid versus Transaxillary Access for Transcatheter Aortic Valve Replacement with a Self-Expanding Valve: A Propensity-Matched Analysis. JTCVS Tech..

[43] Giustino G., Sorrentino S., Mehran R., Faggioni M., Dangas G. (2017). Cerebral Embolic Protection During TAVR. J. Am. Coll. Cardiol..

[44] Miceli A., Murzi M., Gilmanov D., Fugà R., Ferrarini M., Solinas M., Glauber M. (2014). Minimally Invasive Aortic Valve Replacement Using Right Minithoracotomy Is Associated with Better Outcomes than Ministernotomy. J. Thorac. Cardiovasc. Surg..

[45] Taylor M., Low J., Apparau D., Mehta V., Venkateswaran R. (2021). Traversing the Learning Curve Associated with a New Minimal Access Aortic Valve Replacement Service. Braz. J. Cardiovasc. Surg..

[46] Phan K., Xie A., Di Eusanio M., Yan T.D. (2014). A Meta-Analysis of Minimally Invasive Versus Conventional Sternotomy for Aortic Valve Replacement. Ann. Thorac. Surg..

[47] Glauber M., Di Bacco L., Cuenca J., Di Bartolomeo R., Baghai M., Zakova D., Fischlein T., Troise G., Viganò G., Solinas M. (2020). Minimally Invasive Aortic Valve Replacement with Sutureless Valves: Results From an International Prospective Registry. Innovations.

[48] Filip G., Bryndza M.A., Konstanty-Kalandyk J., Piatek J., Wegrzyn P., Ceranowicz P., Brzezinski M., Lakkireddy D., Kapelak B., Bartuś K. (2018). Ministernotomy or Sternotomy in Isolated Aortic Valve Replacement? Early Results. Kardiochirurgia Torakochirurgia Pol..

[49] Mack M.J., Leon M.B., Thourani V.H., Makkar R., Kodali S.K., Russo M., Kapadia S.R., Malaisrie S.C., Cohen D.J., Pibarot P. (2019). Transcatheter Aortic-Valve Replacement with a Balloon-Expandable Valve in Low-Risk Patients. N. Engl. J. Med..

[50] Bourguignon T., Bouquiaux-Stablo A.-L., Candolfi P., Mirza A., Loardi C., May M.-A., El-Khoury R., Marchand M., Aupart M. (2015). Very Long-Term Outcomes of the Carpentier-Edwards Perimount Valve in Aortic Position. Ann. Thorac. Surg..

[51] Elsebaie A., Boutros C.S., Awad A.K., Sanad M., Pelletier M., Abu-Omar Y., El-Diasty M. (2026). Defining the Learning Curve in Minimally Invasive Cardiac Surgery: A Systematic Review and Meta-Analysis. Ann. Thorac. Surg..

[52] Blackman D.J., Saraf S., MacCarthy P.A., Myat A., Anderson S.G., Malkin C.J., Cunnington M.S., Somers K., Brennan P., Manoharan G. (2019). Long-Term Durability of Transcatheter Aortic Valve Prostheses. J. Am. Coll. Cardiol..

[53] Vahanian A., Beyersdorf F., Praz F., Milojevic M., Baldus S., Bauersachs J., Capodanno D., Conradi L., De Bonis M., De Paulis R. (2022). 2021 ESC/EACTS Guidelines for the Management of Valvular Heart Disease: Developed by the Task Force for the Management of Valvular Heart Disease of the European Society of Cardiology (ESC) and the European Association for Cardio-Thoracic Surgery (EACTS). Rev. Española Cardiol..

[54] Siontis G.C.M., Praz F., Pilgrim T., Mavridis D., Verma S., Salanti G., Søndergaard L., Jüni P., Windecker S. (2016). Transcatheter Aortic Valve Implantation vs. Surgical Aortic Valve Replacement for Treatment of Severe Aortic Stenosis: A Meta-Analysis of Randomized Trials. Eur. Heart J..

[55] Head S.J., Milojevic M., Daemen J., Ahn J.-M., Boersma E., Christiansen E.H., Domanski M.J., Farkouh M.E., Flather M., Fuster V. (2018). Mortality after Coronary Artery Bypass Grafting versus Percutaneous Coronary Intervention with Stenting for Coronary Artery Disease: A Pooled Analysis of Individual Patient Data. Lancet.

[56] Solinas M., Bianchi G., Chiaramonti F., Margaryan R., Kallushi E., Gasbarri T., Santarelli F., Murzi M., Farneti P., Leone A. (2020). Right Anterior Mini-Thoracotomy and Sutureless Valves: The Perfect Marriage. Ann. Cardiothorac. Surg..

[57] Popma J.J., Deeb G.M., Yakubov S.J., Mumtaz M., Gada H., O’Hair D., Bajwa T., Heiser J.C., Merhi W., Kleiman N.S. (2019). Transcatheter Aortic-Valve Replacement with a Self-Expanding Valve in Low-Risk Patients. N. Engl. J. Med..

[58] Yoon S.-H., Bleiziffer S., De Backer O., Delgado V., Arai T., Ziegelmueller J., Barbanti M., Sharma R., Perlman G.Y., Khalique O.K. (2017). Outcomes in Transcatheter Aortic Valve Replacement for Bicuspid Versus Tricuspid Aortic Valve Stenosis. J. Am. Coll. Cardiol..

