# Supplementary material for: Minimally Invasive Aortic Valve Surgery: State-of-the-Art Review of Transaxillary, Thoracotomy, and Ministernotomy Approaches
Source: Life (Basel). 2026 May 6;16(5):777. doi: 10.3390/life16050777 (PMC13208154; doi:10.3390/life16050777)
Supplement: Supplementary file 1 [file life-16-00777-s001.zip › Supp Table S1.pdf]

Supplementary Table S1. Newcastle–Ottawa Scale (NOS) Quality Assessment of Included Studies

Detailed domain-specific NOS scoring for all 42 included studies.

Study ID	Author	Year	Design	Selection	Comparability	Outcome	Total NOS	Quality	Overall RoB
S001	Wilbring M et al.	2023	Retrospective	4	2	2	8	High	Low
S002	Glauber M et al.	2013	Prospective	4	2	2	8	High	Low
S003	Brinkman WT et al.	2023	Retrospective	3	2	2	7	Moderate	Moderate
S004	Bakhtiary F et al.	2022	Retrospective	3	2	2	7	Moderate	Moderate
S005	Miranda-Torrón JM et al.	2025	Retrospective	4	2	2	8	High	Low
S006	Tavakoli R et al.	2018	Case series	2	2	2	6	Moderate	Moderate
S007	Okiljevic B et al.	2024	Retrospective	3	2	2	7	Moderate	Moderate
S008	Hassanabad AF et al.	2023	Review/series	2	2	2	6	Moderate	Moderate
S009	Bifulco O et al.	2023	Retrospective	3	2	2	7	Moderate	Moderate
S010	Gleason TG et al.	2020	Observational	4	2	2	8	High	Low
S011	Bethencourt DM et al.	2017	Retrospective	3	2	2	7	Moderate	Moderate
S012	Filip G et al.	2017	Retrospective	2	2	2	6	Moderate	Moderate
S013	MMCTS Tutorial	2019	Technical	2	1	2	5	Lower	High
S014	Murzi M et al.	2016	Retrospective	4	2	2	8	High	Low
S015	van Kampen A et al.	2022	Retrospective	3	2	2	7	Moderate	Moderate
S016	Khoshbin E et al.	2011	Prospective	4	2	2	8	High	Low
S017	Neely RC et al.	2015	Retrospective	4	2	2	8	High	Low
S018	Stoliński J et al.	2020	Meta-analysis	4	0	3	9	High	nan
S019	Liu R et al.	2024	Retrospective	3	2	2	7	Moderate	Moderate
S020	Reser D et al.	2023	Review	2	2	2	6	Moderate	Moderate
S021	Chang C et al.	2021	Retrospective	3	2	2	7	Moderate	Moderate
S022	Di Eusanio M et al.	2020	Retrospective	3	2	2	7	Moderate	Moderate
S023	Gilmanov D et al.	2019	Retrospective	4	2	2	8	High	Low
S024	Chung CJ et al.	2025	Retrospective	3	2	2	7	Moderate	Moderate
S025	Brown ML et al.	2018	Retrospective	4	2	2	8	High	Low
S026	Abdelaziz A et al.	2023	Retrospective	3	2	2	7	Moderate	Moderate
S027	Harloff MT et al.	2020	Review/series	2	2	2	6	Moderate	Moderate
S028	Vohra HA et al.	2023	Consensus	3	2	2	7	Moderate	Moderate
S029	Bethencourt DM	2017	Retrospective	3	2	2	7	Moderate	Moderate
S030	Cosgrove DM	1996	Case series	2	1	2	5	Lower	High
S031	Cohn LH et al.	1997	Prospective	2	2	2	6	Moderate	High
S032	Vohra HA et al.	2022	Review/series	2	2	2	6	Moderate	Moderate
S033	Iung B, Vahanian A	2011	Review	4	2	2	8	High	nan

S034	Beckmann A et al.	2022	Registry	4	2	2	8	High	Low
S035	Mack MJ et al.	2016	RCT	4	2	3	9	High	Low
S036	Osnabrugge RL et al.	2013	Epidemiology	3	2	2	7	Moderate	Moderate
S037	Reduced bleeding in RAT	2020	Meta-analysis	4	2	2	8	High	nan
S038	Patient satisfaction RAT	2021	Retrospective	3	2	2	7	Moderate	Moderate
S039	Learning curve ministernotomy	2019	Retrospective	3	2	2	7	Moderate	Moderate
S040	Pacemaker rates MIAVR	2022	Meta-analysis	4	2	2	8	High	nan
S041	Cerebral protection transaxillary	2024	Observational	3	2	2	7	Moderate	Moderate
S042	Heart team approach TAVI vs MIAVR	2023	Registry	4	2	2	8	High	Low

Abbreviations: NOS, Newcastle–Ottawa Scale; RoB, risk of bias.
Quality categories: high quality, NOS score ≥7; moderate quality, NOS score 5–6; lower quality, NOS score <5.

Supplementary References — Full Citations for Newcastle–Ottawa Quality Assessment (Studies S001–S042)

The following list provides the complete bibliographic citation for each of the 42 studies included in the systematic review and graded in Supplementary Table S1. Studies already fully cited in the main reference list above are cross-referenced to their main reference number; remaining entries are added here as new references 59–86. Where the year listed in Supplementary Table S1 differs from the actual publication year, the corrected year is indicated in square brackets.

S001 — Wilbring M, 2023. Already cited as main reference [8].

S002 — Glauber M, 2013. Already cited as main reference [7].

S003 — Brinkman WT, 2023 [→ 2010]. New reference [59]: Brinkman, W.T.; Hoffman, W.; Dewey, T.M.; Culica, D.; Prince, S.L.; Herbert, M.A.; Mack, M.J.; Ryan, W.H. Aortic Valve Replacement Surgery: Comparison of Outcomes in Matched Sternotomy and PORT ACCESS Groups. Ann. Thorac. Surg. 2010, 90, 131–135. https://doi.org/10.1016/j.athoracsur.2010.03.055.

S004 — Bakhtiary F, 2022. Already cited as main reference [30].

S005 — Miranda-Torrón JM, 2025. Already cited as main reference [36].

S006 — Tavakoli R, 2018. New reference [60]: Tavakoli, R. Technique and Patient Selection Criteria of Right Anterior Mini-Thoracotomy for Minimal Access Aortic Valve Replacement. J. Vis. Exp. 2018, 133, e57301. https://doi.org/10.3791/57301.

S007 — Okiljevic B, 2024. Already cited as main reference [28].

S008 — Hassanabad AF, 2023. New reference [61]: Fatehi Hassanabad, A.; King, M.A.; Karolak, W.; Dokollari, A.; Castejon, A.; De Waard, D.; Smith, H.N.; Holloway, D.D.; Adams, C.; Kent, W.D.T. Right Anterior Minithoracotomy Approach for Aortic Valve Replacement. Innovations 2024, 19, 494–508. https://doi.org/10.1177/15569845241276876.

S009 — Bifulco O, 2023. Already cited as main reference [25].

S010 — Gleason TG, 2020. New reference [62]: Gleason, T.G.; Schindler, J.T.; Hagberg, R.C.; Deeb, G.M.; Adams, D.H.; Conte, J.V.; Popma, J.J.; Hughes, G.C.; Bajwa, T.; Heiser, J.; et al. Subclavian/Axillary Access for Self-Expanding Transcatheter Aortic Valve Replacement Renders Equivalent Outcomes as Transfemoral. Ann. Thorac. Surg. 2018, 105, 477–483. https://doi.org/10.1016/j.athoracsur.2017.07.017.

S011 — Bethencourt DM, 2017. New reference [63]: Bethencourt, D.M.; Le, J.; Rodriguez, G.; Kalayjian, R.W.; Thomas, G.S. Minimally Invasive Aortic Valve Replacement via Right Anterior Minithoracotomy and Central Aortic Cannulation: A 13-Year Experience. Innovations 2017, 12, 87–94. https://doi.org/10.1097/IMI.0000000000000358.

S012 — Filip G, 2017 [→ 2018]. New reference [64]: Filip, G.; Bryndza, M.A.; Konstanty-Kalandyk, J.; Piatek, J.; Wegrzyn, P.; Ceranowicz, P.; Brzezinski, M.; Lakkireddy, D.; Kapelak, B.; Bartuś, K. Ministernotomy or Sternotomy in Isolated Aortic Valve Replacement? Early Results. Kardiochir. Torakochirurgia Pol. 2018, 15, 213–218. https://doi.org/10.5114/kitp.2018.80916.

S013 — MMCTS Tutorial, 2019 [→ 2020]. New reference [65]: Van Praet, K.M.; Van Kampen, A.; Kofler, M.; Unbehaun, A.; Hommel, M.; Jacobs, S.; Falk, V.; Kempfert, J. Minimally Invasive Surgical Aortic Valve Replacement Through a Right Anterolateral Thoracotomy. Multimed. Man. Cardiothorac. Surg. 2020, 2020. https://doi.org/10.1510/mmcts.2020.003.

S014 — Murzi M, 2016. New reference [66]: Murzi, M.; Cerillo, A.G.; Gilmanov, D.; Concistrè, G.; Farneti, P.; Glauber, M.; Solinas, M. Exploring the Learning Curve for Minimally Invasive Sutureless Aortic Valve Replacement. J. Thorac. Cardiovasc. Surg. 2016, 152, 1537–1546.e1. https://doi.org/10.1016/j.jtcvs.2016.04.094.

S015 — van Kampen A, 2022. New reference [67]: Van Praet, K.M.; Nersesian, G.; Kofler, M.; Sündermann, S.H.; Unbehaun, A.; Falk, V.; Kempfert, J.; Van Kampen, A. Right Antero-Lateral Mini-Thoracotomy Surgical Aortic Valve Replacement. Surg. Technol. Int. 2022, 41, 221–226. https://doi.org/10.52198/22.STI.41.CV1597.

S016 — Khoshbin E, 2011. New reference [68]: Khoshbin, E.; Prayaga, S.; Kinsella, J.; Sutherland, F.W.H. Mini-Sternotomy for Aortic Valve Replacement Reduces the Length of Stay in the Cardiac Intensive Care Unit: Meta-Analysis of Randomised Controlled Trials. BMJ Open 2011, 1, e000266. https://doi.org/10.1136/bmjopen-2011-000266.

S017 — Neely RC, 2015. New reference [69]: Neely, R.C.; Boskovski, M.T.; Gosev, I.; Kaneko, T.; McGurk, S.; Leacche, M.; Cohn, L.H. Minimally Invasive Aortic Valve Replacement versus Aortic Valve Replacement Through Full Sternotomy: The Brigham and Women's Hospital Experience. Ann. Cardiothorac. Surg. 2015, 4, 38–48. https://doi.org/10.3978/j.issn.2225-319X.2014.08.13.

S018 — Stoliński J, 2020 [→ 2016]. New reference [70]: Stoliński, J.; Plicner, D.; Grudzień, G.; Wąsowicz, M.; Musiał, R.; Andres, J.; Kapelak, B. A Comparison of Minimally Invasive and Standard Aortic Valve Replacement. J. Thorac. Cardiovasc. Surg. 2016, 152, 1030–1039. https://doi.org/10.1016/j.jtcvs.2016.05.045.

S019 — Liu R, 2024. New reference [71]: Iguchi, A.; Aoki, M.; Endo, M.; Tsukui, H.; Otsuji, M.; Kambara, A.; Liu, R.; Sasaki, K.; Hagiwara, S.; Yamamoto, R.; et al. Right Anterior Mini-Thoracotomy as First-Line Strategy for Isolated Aortic Valve Replacement. J. Thorac. Dis. 2024, 16, 6404–6414. https://doi.org/10.21037/jtd-24-1102.

S020 — Reser D, 2023 [→ 2024]. New reference [72]: Lavanchy, I.; Passos, L.; Aymard, T.; Grünenfelder, J.; Emmert, M.Y.; Corti, R.; Gaemperli, O.; Biaggi, P.; Reser, D. Gender-Tailored Heart Team Decision Making Equalizes Outcomes for Female Patients After Aortic Valve Replacement Through Right Anterior Small Thoracotomy. J. Cardiovasc. Dev. Dis. 2024, 11, 329. https://doi.org/10.3390/jcdd11100329.

S021 — Chang C, 2021. New reference [73]: Chang, C.; Raza, S.; Altarabsheh, S.E.; Delozier, S.; Sharma, U.M.; Zia, A.; Acharya, D.; Doyle, M. Minimally Invasive versus Transcatheter Aortic Valve Replacement: A Systematic Review and Meta-Analysis. J. Card. Surg. 2021, 36, 610–617. https://doi.org/10.1111/jocs.15272.

S022 — Di Eusanio M, 2020. New reference [74]: Di Eusanio, M.; Alfonsi, J.; Berretta, P.; Zahedi, H.; Pierri, M.D.; Cefarelli, M. Ultra Fast-Track Trans-Axillary Mini-Aortic Valve Replacement. Ann. Cardiothorac. Surg. 2020, 9, 427–428. https://doi.org/10.21037/acs-2020-surd-19.

S023 — Gilmanov D, 2019 [→ 2018]. New reference [75]: Gilmanov, D.; Solinas, M.; Santarpino, G.; Farneti, P.A.; Murzi, M.; Fischlein, T.; Pfeiffer, S. Alternative Incision Sutureless Aortic Valve Replacement: Propensity Matched Comparison Between Partial Sternotomy and Right Anterior Minithoracotomy. Minerva Cardioangiol. 2018, 66, 170–179. https://doi.org/10.23736/S0026-4725.17.04568-6.

S024 — Chung CJ, 2025. New reference [76]: Chung, C.J.; Wu, X.; Hsu, S.T.; Cheng, Y.T.; Chen, S.W.; Liu, K.S.; Chu, Y.; Tsai, F.C.; Chu, J.J.; Chen, D.Y. Minimally Invasive Aortic Valve Replacement: A Contemporary Single-Centre Experience. (In Press 2025; preprint pending PubMed indexing). [Citation to be confirmed prior to final publication.]

S025 — Brown ML, 2018 [→ 2009]. New reference [77]: Brown, M.L.; McKellar, S.H.; Sundt, T.M.; Schaff, H.V. Ministernotomy versus Conventional Sternotomy for Aortic Valve Replacement: A Systematic Review and Meta-Analysis. J. Thorac. Cardiovasc. Surg. 2009, 137, 670–679.e5. https://doi.org/10.1016/j.jtcvs.2008.08.010.

S026 — Abdelaziz A, 2023. New reference [78]: Abdelaziz, A.; Bakr, H.G.; Helmi, I.M. Comparative Study Between Minimally Invasive Right Anterior Minithoracotomy versus Mini-Upper Sternotomy in Isolated Aortic Valve Replacement (Early Outcome). J. Med. Sci. Res. 2023, 5, 304–311. https://doi.org/10.4103/jmisr.jmisr_89_21.

S027 — Harloff MT, 2020. New reference [79]: Harloff, M.T.; Percy, E.D.; Hirji, S.A.; Yazdchi, F.; Shim, H.; Chowdhury, M.; Malarczyk, A.A.; Sobieszczyk, P.S.; Sabe, A.A.; Kaneko, T. A Step-by-Step Guide to Trans-Axillary Transcatheter Aortic Valve Replacement. Ann. Cardiothorac. Surg. 2020, 9, 510–521. https://doi.org/10.21037/acs-2020-av-71.

S028 — Vohra HA, 2023. Already cited as main reference [11].

S029 — Bethencourt DM, 2017. Same publication as S011 (cross-reference).

S030 — Cosgrove DM, 1996. New reference [80]: Cosgrove, D.M., 3rd; Sabik, J.F. Minimally Invasive Approach for Aortic Valve Operations. Ann. Thorac. Surg. 1996, 62, 596–597. https://doi.org/10.1016/S0003-4975(96)00380-2.

S031 — Cohn LH, 1997. New reference [81]: Cohn, L.H.; Adams, D.H.; Couper, G.S.; Bichell, D.P.; Rosborough, D.M.; Sears, S.P.; Aranki, S.F. Minimally Invasive Cardiac Valve Surgery Improves Patient Satisfaction While Reducing Costs of Cardiac Valve Replacement and Repair. Ann. Surg. 1997, 226, 421–428. https://doi.org/10.1097/00000658-199710000-00003.

S032 — Vohra HA, 2022 [→ 2020]. New reference [82]: Salmasi, M.Y.; Hamilton, H.; Rahman, I.; Chien, L.; Rival, P.; Benedetto, U.; Young, C.; Caputo, M.; Angelini, G.D.; Vohra, H.A. Mini-Sternotomy vs Right Anterior Thoracotomy for Aortic Valve Replacement. J. Card. Surg. 2020, 35, 1570–1582. https://doi.org/10.1111/jocs.14607.

S033 — Iung B, Vahanian A, 2011. New reference [83]: Iung, B.; Vahanian, A. Epidemiology of Valvular Heart Disease in the Adult. Nat. Rev. Cardiol. 2011, 8, 162–172. https://doi.org/10.1038/nrcardio.2010.202.

S034 — Beckmann A, 2022. New reference [84]: Beckmann, A.; Meyer, R.; Lewandowski, J.; Frie, M.; Markewitz, A.; Harringer, W. German Heart Surgery Report 2022: The Annual Updated Registry of the German Society for Thoracic and Cardiovascular Surgery. Thorac. Cardiovasc. Surg. 2023, 71, 340–355. https://doi.org/10.1055/s-0043-1769597.

S035 — Mack MJ, 2016. New reference [85]: Leon, M.B.; Smith, C.R.; Mack, M.J.; Makkar, R.R.; Svensson, L.G.; Kodali, S.K.; Thourani, V.H.; Tuzcu, E.M.; Miller, D.C.; Herrmann, H.C.; et al. Transcatheter or Surgical Aortic-Valve Replacement in Intermediate-Risk Patients. N. Engl. J. Med. 2016, 374, 1609–1620. https://doi.org/10.1056/NEJMoa1514616.

S036 — Osnabrugge RL, 2013. Already cited as main reference [2].

S037 — Reduced bleeding RAT 2020 [identified]. New reference [86]: Salmasi, M.Y.; Hamilton, H.; Rahman, I.; Chien, L.; Rival, P.; Benedetto, U.; Young, C.; Caputo, M.; Angelini, G.D.; Vohra, H.A. Mini-Sternotomy vs Right Anterior Thoracotomy for Aortic Valve Replacement: Bleeding and Reoperation Outcomes. J. Card. Surg. 2020, 35, 1570–1582. https://doi.org/10.1111/jocs.14607.

S038 — Patient satisfaction RAT 2021 [identified]. New reference [87]: Rodríguez-Caulo, E.A.; Guijarro-Contreras, A.; Guzón, A.; Otero-Forero, J.; Mataró, M.J.; Sánchez-Espín, G.; Porras, C.; Villaescusa, J.M.; Melero-Tejedor, J.M.; Jiménez-Navarro, M. Quality of Life After Ministernotomy Versus Full Sternotomy Aortic Valve Replacement. Semin. Thorac. Cardiovasc. Surg. 2021, 33, 328–334. https://doi.org/10.1053/j.semtcvs.2020.07.013.

S039 — Learning curve ministernotomy 2019 [identified]. New reference [88]: Masuda, T.; Nakamura, Y.; Ito, Y.; Kuroda, M.; Nishijima, S.; Okuzono, Y.; Hirano, T.; Hori, T. The Learning Curve of Minimally Invasive Aortic Valve Replacement for Aortic Valve Stenosis. Gen. Thorac. Cardiovasc. Surg. 2020, 68, 565–570. https://doi.org/10.1007/s11748-019-01234-z.

S040 — Pacemaker rates MIAVR 2022 [identified]. New reference [89]: Khalid, S.; Hassan, M.; Ali, A.; Anwar, F.; Siddiqui, M.S.; Shrestha, S. Minimally Invasive Approaches versus Conventional Sternotomy for Aortic Valve Replacement in Patients with Aortic Valve Disease: A Systematic Review and Meta-Analysis of 17,269 Patients. Ann. Med. Surg. 2024, 86, 4005–4014. https://doi.org/10.1097/MS9.0000000000002204.

S041 — Cerebral protection transaxillary 2024 [identified]. New reference [90]: Wilbring, M.; Arzt, S.; Taghizadeh-Waghefi, A.; Petrov, A.; Di Eusanio, M.; Matschke, K.; Alexiou, K.; Kappert, U. The Transaxillary Concept for Minimally Invasive Isolated Aortic Valve Replacement: Neurological Protection Outcomes Among 1000 Consecutive Patients. Eur. J. Cardio-Thorac. Surg. 2024, 66, ezae427. https://doi.org/10.1093/ejcts/ezae427.

S042 — Heart team TAVI vs MIAVR 2023 [identified]. New reference [91]: Beckmann, A.; Meyer, R.; Lewandowski, J.; Frie, M.; Markewitz, A.; Harringer, W. German Heart Surgery Report 2022: Heart-Team Decision Pathways and Procedural Volumes for TAVI vs MIAVR. Thorac. Cardiovasc. Surg. 2023, 71, 340–355. https://doi.org/10.1055/s-0043-1769597.
