# Supplementary material for: Minimally Invasive Aortic Valve Surgery: State-of-the-Art Review of Transaxillary, Thoracotomy, and Ministernotomy Approaches
Source: Life (Basel). 2026 May 6;16(5):777. doi: 10.3390/life16050777 (PMC13208154; doi:10.3390/life16050777)
Supplement: Supplementary file 1 [file life-16-00777-s001.zip › Supp Table S2.pdf]

Supplementary Table S2. Operative Characteristics and Technical Details by Approach

Operative Parameter	Transaxillary Access	RAT	Ministernotomy
**INCISION CHARACTERISTICS**			
Skin incision length (cm), mean±SD	5.2±1.1	6.1±0.9	8.2±1.5
Incision location	Anterior axillary fold	2nd–3rd ICS laterally	Midline, upper sternum
Skin-to-first-incision time (min)	3–5	3–5	3–5
Sternal division	None	None	Partial (cranial 1/3 to 1/2)
Sternal division length (cm)	N/A	N/A	6.2±1.8
Rib division	No	Yes (3rd–4th rib)	No
Internal mammary artery preservation	N/A	Preserved (>95%)	Preserved (>99%)
**CANNULATION STRATEGY**			
Arterial cannulation site	Axillary artery (surgical/percutaneous)	Aorta (direct)	Aorta (direct)
Arterial cannula size (Fr)	15–17	18–20	18–20
Venous cannulation (single)	Femoral vein	Right atrium (direct)	Right atrium (direct)
Venous cannulation (two-stage)	Rare	SVC + IVC direct	SVC + IVC direct
Cannula-to-aorta distance (cm)	30–45 (remote)	5–10 (close)	5–15 (variable)
Aortic cross-clamp application	Transesophageal (blind/echo-guided)	Direct visualization	Direct visualization
**VALVE PROSTHESIS SELECTION**			

Rapid deployment valves (%), n	1,720 (79.8)	1,957 (40.0)	2,482 (30.0)
Sutured biological prosthesis (%)	327 (15.2)	2,453 (50.2)	4,968 (60.0)
Mechanical prosthesis (%)	109 (5.1)	482 (9.9)	830 (10.0)
Sutureless prosthesis (%)	NR	150–245 (3.0–5.0)	245–414 (3.0–5.0)
**PROSTHESIS SIZE DISTRIBUTION**			
Valve size 19 mm, n (%)	108 (5.0)	245 (5.0)	414 (5.0)
Valve size 21 mm, n (%)	646 (30.0)	1,467 (30.0)	2,484 (30.0)
Valve size 23 mm, n (%)	1,078 (50.0)	2,453 (50.2)	4,140 (50.0)
Valve size 25 mm, n (%)	324 (15.0)	728 (14.9)	1,242 (15.0)
Mean prosthesis size (mm), mean±SD	22.8±1.4	22.9±1.3	22.7±1.5
**OPERATIVE TIMES AND DURATIONS**			
Total operative time (min), mean±SD	183.4±44.2	145.8±38.5	192.6±51.3
Skin-to-skin time (min), mean±SD	120.0±31.5	65–72 (NR separate)	138.0±34.2
CPB time (min), mean±SD	68.5±22.1	68.3±21.5	76.8±25.4
Aortic cross-clamp time (min), mean±SD	45.8±15.2	47.6±16.1	55.2±18.9
CPB time range (min)	63–75	64–72	74–80
X-clamp time range (min)	41–50	43–52	52–58
**INTRAOPERATIVE MANAGEMENT**			

Transesophageal echocardiography (%), n	2,156 (100)	4,892 (100)	8,280 (100)
Continuous neurological monitoring	538 (25.0)	489 (10.0)	414 (5.0)
Cerebral oximetry (NIRS)	539 (25.0)	293 (6.0)	83 (1.0)
Minimally invasive extracorporeal perfusion	323 (15.0)	1,467 (30.0)	2,484 (30.0)
Cell salvage (autologous)	1,292 (60.0)	3,903 (79.8)	6,624 (80.0)
Antifibrinolytic use (tranexamic acid)	1,505 (69.8)	3,903 (79.8)	6,624 (80.0)
**CONVERSION TO FULL STERNOTOMY**			
Overall conversion rate (%), n	54 (2.5)	196 (4.0)	331 (4.0)
Conversion in first 50 cases (%)	10–15 (2.0–3.0)	20–30 (4.0–6.0)	8–12 (1.2–1.8)
Conversion in cases >100 (%)	0–1 (0–0.5)	2–4 (0.4–0.8)	8–15 (0.1–0.2)
**Reasons for conversion:**			
- Technical difficulty/inadequate exposure	32 (1.5)	98 (2.0)	165 (2.0)
- Intraoperative bleeding	11 (0.5)	49 (1.0)	83 (1.0)
- Cardiac arrest/hemodynamic instability	5–11 (0.2–0.5)	25–49 (0.5–1.0)	41–83 (0.5–1.0)
- Endocarditis/valve destruction	0–3 (0–0.14)	5–10 (0.1–0.2)	10–25 (0.12–0.3)

- Aortic dissection/rupture	0–2 (0–0.09)	5–10 (0.1–0.2)	5–15 (0.06–0.2)
**INTRAOPERATIVE COMPLICATIONS**			
Aortic dissection	0–2 (0–0.09)	0–2 (0–0.04)	0–2 (0–0.02)
Mechanical valve malposition	2–3 (0.09–0.14)	2–3 (0.04–0.06)	4–5 (0.05–0.06)
Left main coronary ostial obstruction	0–2 (0–0.09)	2–3 (0.04–0.06)	5–8 (0.06–0.1)
Ventricular fibrillation	2–8 (0.09–0.37)	5–15 (0.1–0.3)	16–33 (0.2–0.4)
Severe bleeding (requiring transfusion ≥4 units)	8–27 (0.37–1.3)	25–98 (0.5–2.0)	83–166 (1.0–2.0)

Supplementary Table S2: Operative characteristics and technical details by MIAVR approach. Data compiled from 42 studies (2010–2025). Abbreviations: RAT, right anterior thoracotomy; ICS, intercostal space; Fr, French size; SVC, superior vena cava; IVC, inferior vena cava; CPB, cardiopulmonary bypass; NIRS, near-infrared spectroscopy; NR, not reported; N/A, not applicable.
