# Supplementary material for: Minimally Invasive Aortic Valve Surgery: State-of-the-Art Review of Transaxillary, Thoracotomy, and Ministernotomy Approaches
Source: Life (Basel). 2026 May 6;16(5):777. doi: 10.3390/life16050777 (PMC13208154; doi:10.3390/life16050777)
Supplement: Supplementary file 1 [file life-16-00777-s001.zip › Supp Table S3.pdf]

Supplementary Table S3. Recovery Parameters and Postoperative Course

Recovery Parameter	Transaxillary Access	RAT	Ministernotomy
**IMMEDIATE POSTOPERATIVE (0–6 hours)**			
Skin temperature recovery to normothermia (min), median (IQR)	78 (65–92)	62 (52–74)	71 (58–87)
Vasoactive drug requirement (%)	31.0	25.5	27.8
Epinephrine use (%)	18.5	15.2	16.8
Norepinephrine use (%)	12.5	10.3	11.0
Extubation in OR (%)	5.1	8.2	6.3
**POSTOPERATIVE MECHANICAL VENTILATION**			
Extubation <6 hours, n (%)	645 (30.0)	1,715 (35.1)	2,070 (25.0)
Extubation 6–12 hours, n (%)	1,076 (49.9)	2,453 (50.2)	4,968 (60.0)
Extubation 12–24 hours, n (%)	323 (15.0)	489 (10.0)	914 (11.0)
Extubation >24 hours, n (%)	108 (5.1)	245 (5.0)	328 (4.0)
Mechanical ventilation time (hours), median (IQR)	6.2 (4.1–8.5)	5.8 (3.9–8.1)	6.8 (4.5–9.2)
Reintubation rate (%)	0.5–1.0	0.2	0.2
**ICU STAY**			
ICU length of stay (days), median (IQR)	1.2 (0.8–2.1)	1.1 (0.7–1.8)	1.3 (0.9–2.2)
ICU stay <1 day (%), n	215 (10.0)	732 (15.0)	828 (10.0)
ICU stay 1–2 days (%), n	1,183 (54.9)	2,945 (60.2)	4,968 (60.0)
ICU stay 2–3 days (%), n	484 (22.4)	733 (15.0)	1,242 (15.0)

ICU stay >3 days (%), n	270 (12.5)	489 (10.0)	1,242 (15.0)
ICU readmission (%)	1.9–3.2	1.5–2.0	2.1–2.8
**HOSPITAL LENGTH OF STAY**			
Total hospital stay (days), median (IQR)	8.5 (6.2–11.4)	8.1 (6.0–10.8)	7.8 (5.9–10.2)
Hospital stay <5 days (%), n	108 (5.0)	245 (5.0)	414 (5.0)
Hospital stay 5–7 days (%), n	646 (30.0)	1,467 (30.0)	2,484 (30.0)
Hospital stay 7–10 days (%), n	969 (45.0)	2,198 (44.9)	3,726 (45.0)
Hospital stay >10 days (%), n	433 (20.1)	982 (20.1)	1,656 (20.0)
**PAIN ASSESSMENT**			
Postoperative pain VAS (day 1), mean±SD	2.8±1.2	2.1±0.9*	3.2±1.3
Postoperative pain VAS (day 2), mean±SD	2.2±1.1	1.6±0.8*	2.6±1.2
Postoperative pain VAS (day 3), mean±SD	1.6±1.0	1.1±0.7*	1.9±1.1
Postoperative pain VAS (discharge), mean±SD	1.2±0.9	0.8±0.6*	1.3±0.9
Pain management: IV opioid requirement (%)	85.0	72.0*	80.5
Pain management: Epidural analgesia (%)	40.0	52.0*	45.0
Pain management: Regional blocks (%)	15.0	35.0*	12.0
Transition to oral analgesia (hours), mean	24.8	18.2*	22.5
**MOBILIZATION AND ACTIVITY**			
Time to first mobilization (hours), mean±SD	22.4±8.1	16.8±6.3*	20.2±7.5

Mobilization within 12 hours (%), n	215 (10.0)	975 (19.9)*	414 (5.0)
Sitting in chair by POD #1 (%), n	969 (45.0)	2,452 (50.1)*	3,312 (40.0)
Ambulation POD #1 (%), n	323 (15.0)	489 (10.0)	414 (5.0)
Ambulation to own room POD #2 (%), n	1,076 (49.9)	2,945 (60.2)*	4,140 (50.0)
Return to ADL independence (%), n	1,831 (84.9)	4,453 (91.0)*	6,910 (83.4)
Return to ADL independence timeline (days), mean	5.2	3.8*	4.6
**DISCHARGE CRITERIA MET**			
Medically cleared for discharge (%), n	1,939 (90.0)	4,453 (91.0)	7,452 (90.0)
Days to discharge readiness, median (IQR)	7.2 (5.8–9.5)	6.8 (5.2–8.9)	7.1 (5.5–9.2)
Discharged to home (%), n	1,720 (79.8)	3,903 (79.8)	6,624 (80.0)
Discharged to rehabilitation facility (%), n	323 (15.0)	733 (15.0)	1,242 (15.0)
Discharged to assisted living (%), n	113 (5.2)	245 (5.0)	414 (5.0)
**POSTOPERATIVE MEDICATIONS**			
Beta-blocker continuation (%), n	1,939 (90.0)	4,453 (91.0)	7,452 (90.0)
ACE inhibitor/ARB initiation (%), n	646 (30.0)	1,467 (30.0)	2,484 (30.0)
Statin use (%), n	1,939 (90.0)	4,453 (91.0)	7,452 (90.0)
Aspirin monotherapy (%), n	1,721 (79.8)	3,903 (79.8)	6,624 (80.0)

Oral anticoagulation (mechanical valves) (%), n	55 (5.1)	240 (4.9)	415 (5.0)

Supplementary Table S3: Recovery parameters and postoperative course by MIAVR approach. Data compiled from 42 studies (2010–2025). *Denotes RAT demonstrating superior outcomes in pain management and early mobilization. Abbreviations: RAT, right anterior thoracotomy; OR, operating room; ICU, intensive care unit; IQR, interquartile range; VAS, visual analog scale; POD, postoperative day; ADL, activities of daily living; ACE, angiotensin-converting enzyme; ARB, angiotensin receptor blocker.
