# Supplementary material for: Minimally Invasive Aortic Valve Surgery: State-of-the-Art Review of Transaxillary, Thoracotomy, and Ministernotomy Approaches
Source: Life (Basel). 2026 May 6;16(5):777. doi: 10.3390/life16050777 (PMC13208154; doi:10.3390/life16050777)
Supplement: Supplementary file 1 [file life-16-00777-s001.zip › Supp Table S4.pdf]

Supplementary Table S4. Long-Term Outcomes and Valve Durability (1–20 Year Follow-up)

Outcome Parameter	Transaxillary Access	RAT	Ministernotomy
**SURVIVAL**			
30-day survival (%), n	2,12–2,13 (98.5–99.1)	4,79–4,85 (98.0–99.1)	8,09–8,25 (97.8–99.6)
1-year survival (%), n	2,09–2,12 (96.8–98.4)	4,50–4,80 (94.0–98.0)	7,86–8,18 (94.9–98.7)
3-year survival (%), n	1,97–1,97 (91.2–91.3)	4,33–4,60 (88.6–94.0)	7,49–7,67 (90.4–92.5)
5-year survival (%), n	NR	4,54–4,71 (92.7–96.3)	6,97–7,25 (84.2–87.5)
10-year survival (%), n	NR	3,94–4,17 (80.5–85.2)	6,03–6,48 (72.8–78.3)
20-year survival (%), n	NR	~1,96 (40)	NR
**VALVE-RELATED COMPLICATIONS (Long-term)**			
Endocarditis, n (%)	NR	44–49 (0.9–1.0)	83–166 (1.0–2.0)
**REOPERATION/REINTERVENTION**			
Unplanned reoperation (all causes), n (%)	NR	93–147 (1.9–3.0)	166–332 (2.0–4.0)
Valve-related reoperation, n (%)	NR	~49 (1.0)	~83 (1.0)
Freedom from reoperation (10 years), %	NR	95.8	94.5
**Reoperation indications:**			
- Endocarditis	NR	~10 (0.2)	~16 (0.2)
- Paravalvular leak	NR	~10 (0.2)	~16 (0.2)
- Pannus/thrombosis	NR	~15 (0.3)	~25 (0.3)

- Bleeding (anticoagulation)	NR	~7 (0.14)	~12 (0.15)
- Other valve-related	NR	~7 (0.14)	~14 (0.17)
**QUALITY OF LIFE AND FUNCTIONAL OUTCOMES**			
Return to work (%), n	NR	3,66–3,90 (74.8–79.8)	6,14–6,62 (74.2–80.0)
Time to return to work (weeks), median	NR	8–12	10–14
Return to exercise/sports (%), n	NR	3,90–4,45 (79.8–91.0)	5,31–6,62 (64.1–80.0)
Return to normal activities of living (%), n	NR	4,45–4,82 (91.0–98.5)	6,62–7,45 (80.0–90.0)
Improvement in NYHA functional class (%), n	NR	4,45–4,82 (91.0–98.5)	6,62–7,45 (80.0–90.0)
NYHA class I–II at follow-up (%), n	NR	3,90–4,45 (79.8–91.0)	5,31–6,62 (64.1–80.0)
**CARDIOVASCULAR EVENTS (Cumulative)**			
Myocardial infarction, n (%)	NR	~20 (0.4)	~41 (0.5)
Stroke/TIA (late), n (%)	NR	~20 (0.4)	~41 (0.5)
Arrhythmia requiring intervention, n (%)	NR	~98 (2.0)	~166 (2.0)
Heart failure (new/worsening), n (%)	NR	~147–196 (3.0–4.0)	~249–332 (3.0–4.0)
Major adverse cardiovascular event (MACE), n (%)	NR	~196–245 (4.0–5.0)	~331–414 (4.0–5.0)
**COSMETIC SATISFACTION (Long-term)**			
Patient satisfaction with scar (% excellent)	~90	96	~75
Scar visibility concern (% not concerned)	~85	92	65
Aesthetic outcome satisfaction (%), n	NR	4,45–4,89 (91.0–98.5)	5,31–6,62 (64.1–80.0)

Cosmesis-related self-esteem improvement (%), n	NR	3,90–4,45 (79.8–91.0)	2,48–4,14 (30.0–50.0)

Table S4: Long-term outcomes and valve durability (1–20 year follow-up) by MIAVR approach. Data compiled from studies with extended follow-up periods. RAT demonstrates excellent 10-year survival (80.5–85.2%) and freedom from reoperation (95.8%). Abbreviations: RAT, right anterior thoracotomy; NYHA, New York Heart Association; TIA, transient ischemic attack; MACE, major adverse cardiovascular event; NR, not reported.
