# Supplementary material for: Minimally Invasive Aortic Valve Surgery: State-of-the-Art Review of Transaxillary, Thoracotomy, and Ministernotomy Approaches
Source: Life (Basel). 2026 May 6;16(5):777. doi: 10.3390/life16050777 (PMC13208154; doi:10.3390/life16050777)
Supplement: Supplementary file 1 [file life-16-00777-s001.zip › Supp Table S5.pdf]

Supplementary Table S5. Risk Stratification Outcomes - EuroSCORE II Risk Groups

EuroSCORE II Risk Group	Low Risk (≤2%)	Intermediate Risk (2–5%)	High Risk (5–10%)	Very High Risk (>10%)
**PATIENT DISTRIBUTION BY APPROACH**				
**Transaxillary (n=2,156)**				
Number of patients, n (%)	86 (4.0)	1,075 (49.9)	753 (34.9)	242 (11.2)
30-day mortality (%)	0.0	0.9	3.2	8.3
Stroke rate (%)	1.2	2.1	5.4	8.9
Bleeding revision (%)	1.2	2.4	4.1	6.2
**RAT (n=4,899)**				
Number of patients, n (%)	489 (10.0)	2,945 (60.2)	1,220 (24.9)	245 (5.0)
30-day mortality (%)	0.0	0.6	1.4	4.9
Stroke rate (%)	0.2	0.5	1.1	3.3
Bleeding revision (%)	1.2	2.0	3.5	5.3
**Ministernotomy (n=8,280)**				
Number of patients, n (%)	1,656 (20.0)	4,968 (60.0)	1,242 (15.0)	414 (5.0)
30-day mortality (%)	0.0	0.2	0.9	2.9
Stroke rate (%)	0.1	0.6	1.3	2.8
Bleeding revision (%)	2.1	4.5	8.1	11.2

Supplementary Table S5: Risk stratification outcomes stratified by EuroSCORE II risk groups across three MIAVR approaches. Data demonstrates increasing complication rates with higher risk scores across all approaches. Transaxillary cohort included higher proportion of high-risk and very high-risk patients (46.1%) compared to RAT (29.9%) and ministernotomy (20.0%), reflecting patient selection for redo surgery and complex cases. Abbreviations: RAT, right anterior thoracotomy; EuroSCORE II, European System for Cardiac Operative Risk Evaluation.
